# Distribution of Antibiotic-Resistant *Enterobacteriaceae* Pathogens in Potable Spring Water of Eastern Indian Himalayas: Emphasis on Virulence Gene and Antibiotic Resistance Genes in *Escherichia coli*

**DOI:** 10.3389/fmicb.2020.581072

**Published:** 2020-11-05

**Authors:** Ashish Kumar Singh, Saurav Das, Santosh Kumar, Varsha Rani Gajamer, Ishfaq Nabi Najar, Yangchen D. Lepcha, Hare Krishna Tiwari, Samer Singh

**Affiliations:** ^1^Department of Microbiology, School of Life Sciences, Sikkim University, Gangtok, India; ^2^Department of Agronomy and Horticulture, University of Nebraska–Lincoln, Lincoln, NE, United States; ^3^State Institute of Rural Development (SIRD), Government of Sikkim, Gangtok, India; ^4^Centre of Experimental Medicine and Surgery (CEMS), Institute of Medical Sciences, Banaras Hindu University, Varanasi, India

**Keywords:** spring, *Enterobacteriaceae*, virulence gene, antibiotic resistance gene, multidrug resistant (MDR), water quality, waterborne pathogens

## Abstract

Every year millions of people die due to fatal waterborne diseases around the world especially in developing countries like India. Sikkim, a northeastern state of India, greatly depends on natural water sources. About 80% of the population of Sikkim depends on natural spring water for domestic as well as agricultural use. Recent waterborne disease outbreaks in the state raises a concerning question on water quality. In this study, we analyzed water quality especially for the detection of *Enterobacteriaceae* members from four districts of the state. Isolation with selective culture media techniques and taxonomic characterization of *Enterobacteriaceae* bacteria with 16S rRNA gene showed the prevalence of *Escherichia coli* (37.50%), *Escherichia fergusonii* (29.41%), *Klebsiella oxytoca* (36.93%), *Citrobacter freundii* (37.92%), *Citrobacter amalonaticus* (43.82%), *Enterobacter sp.* (43.82%), *Morganella morganii* (43.82%), *Hafnia alvei* (32.42%), *Hafnia paralvei* (38.74%), and *Shigella flexneri* (30.47%) in the spring water of Sikkim. Antibiotic susceptibility test (AST) showed resistance of the isolates to common antibiotics like ampicillin, amoxicillin as well as to third generation antibiotics like ceftazidime and carbapenem. None of the isolates showed resistance to chloramphenicol. *E. coli* isolated from spring water of Sikkim showed presence of different virulence genes such as *stx1* (81.81%), *elt* (86.66%), and *eae* (66.66%) along with resistance gene for ampicillin (*CITM*) (80%), quinolones (*qnrB*) (44.44%), tetracycline (*tetO*) (66.66%), and streptomycin (*aadA1*) (66.66%). The data indicates a high incidence rate of multiple antibiotic resistant enteric bacteria in the spring water of Sikkim. Additionally, the presence of enteric bacteria in the water samples indicates widespread fecal contamination of the spring water.

## Introduction

Water is important for every facet of life (Mishra et al., 2018). Surface water acts as a natural source of freshwater and is important for drinking, cooking as well as other household purposes (Vaz-Moreira et al., 2013). Approximately one-third of global freshwater reserves are subsurface streams (Hemme et al., 2015). Safe and clean drinking water is a basic right to every human being for a healthy life. Globally freshwater is becoming a limited resource due to population expansion, anthropoegenic contamination and climate change (Hassan Rashid et al., 2018). Contaminated water can cause several illnesses including dysentery, cholera, diarrhea, typhoid, and in chronic cases leading to loss of life on a normal basis especially in developing countries. Due to lack of quality health care systems and insufficiency in the supply of pure potable water, developing countries are on the alarming list of waterborne disease outbreaks (World Health Organisation, 2018). It is reported that 80% of the diseases worldwide occurs due to contaminated water and water borne pathogens (Haseena et al., 2017). Diarrhea alone is the cause of 8,42,000 deaths every year and by 2025 half of the world is expected to be living in the water stressed areas increasing the risks of dying from waterborne diseases (World Health Organisation, 2018).

Antibiotic resistance is responsible for thousands of death annually and which is projected to increase dramatically as a global health hazard (Momtaz et al., 2013; Adzitey, 2020). It is estimated that antibiotic resistance might lead to ten million deaths annually by 2050 (Bengtsson-Palme et al., 2018; Praveenkumarreddy et al., 2020). Treatment of infectious diseases has been revolutionized since the discovery, production and use of antimicrobial substances (Stange et al., 2016). However, the misuse of antimicrobial substances are the major cause of emergence of antibiotic resistance in pathogenic bacteria (Singh et al., 2018a; Meng et al., 2020). Most of the antibiotics provided to humans for household purpose ends up in the sewage system from excretion and finally to water sources (Karkman et al., 2018). Studies reported that after consumption of drugs, a significant portion (50 – 90%) of antibiotics excreted by humans in domestic sewage systems remains unchanged keeping its chemical properties sufficient to meet the therapeutic use (Costa and Arpini, 2016). Chemical properties of drugs poses a potential risk to public health and environment as their resistant components are difficult to decompose (Costa and Arpini, 2016). Our ecosystem is constantly exposed to a wide variety of antimicrobials through wastewater treatment plants, agricultural runoff as well as animal-related and anthropogenic bustle (Baquero et al., 2008; Anand et al., 2016). The sewage treatment plants contain a large number of antibiotic resistant bacteria (ARB) with antibiotic resistance genes (ARGs) which get discharged into water bodies. Use of untreated water from these bodies, increases the probability of public health risks due to them acting as a source of ARB and ARGs (Stange et al., 2016; Bengtsson-Palme et al., 2018; Obayiuwana and Ibekwe, 2020). Contaminated water provides a perfect selective and natural media for interaction between ARB and environmental bacteria for the horizontal shift of ARGs (Anand et al., 2016). The presence of ARGs in human pathogens imposes a negative effect on health including failure of treatments, prolonged treatment periods, and in chronic cases deaths (Bueno et al., 2018). It is reported that about 7,00,000 peoples die each year due to the infection of ARB and that may increase up to 10,000,000 peoples per year by 2050 (Na et al., 2018; Morris and Cerceo, 2020).

Water quality assessment is the need of this hour to predict and minimize the future risks of water-borne diseases especially in rural areas of developing countries. Commensal bacteria living in the gastrointestinal tract of humans and animals mostly from *Enterobacteriaceae* family are used as a microbiological indicator of water quality and fecal contamination (Stange et al., 2016; Adegoke et al., 2020). There are several common fecal coliform genera such as *Escherichia, Klebsiella, Citrobacter, Enterobacter, Morganella*, and *Hafnia* (Stange et al., 2016). *Escherichia coli* is a key member of the *Enterobacteriaceae* family, lives commensally in the gastrointestinal tract without causing any infections. However, acquisition of virulence gene (VG) and ARGs by *E. coli* can cause a wide range of intestinal and extra-intestinal infections including diarrhea, urinary tract infection, and meningitis (Stange et al., 2016; Subbiah et al., 2020). Emergence and spreading of new antibiotic resistance in the *Enterobacteriaceae* family is becoming a major risk to clinical facilities to treat common infections. Without a comprehensive understanding of the future health risk, it can put us back into the pre-antibiotic era where common infections and minor injuries can become fatal again.

Sikkim (27° 05′ to 28° 07′ N latitudes and 87° 59′ to 88° 56′ E longitudes), an Eastern Himalayan state of India, lies between Nepal and Bhutan (Tambe et al., 2013). The state faces a tremendous scarcity of potable and treated drinking water for the rural as well as urban population. Though the Himalayan range is a source of countless perennial rivers, Sikkimese people largely depend on spring water for their sustenance. The mountain springs, locally known as “*Dharas*,” are the natural discharges of groundwater from various aquifers (Tambe et al., 2012, 2013). About 80% of the total rural community is solely dependent on spring water for their domestic and potable purpose (Tambe et al., 2012; Singh et al., 2018a, 2019). Sikkimese people use untreated spring water for their daily uses which is a major threat to public health, as these could serve as a reservoir of waterborne pathogens (Tambe et al., 2013). The dependence of the people of the state on spring water solely defines the importance of this study. This study reports the distribution of Gram-negative bacteria in spring water, and their VGs, and ARGs from four districts *viz.* East (E), West (W), South (S), and North (N). Lack of such previous reports makes it an important foundation for structuring and devising the future water treatment protocols and for revising and restructuring the government policies to tackle future health risks.

## Materials and Methods

### Description of Study Site

**FIGURE 1:**
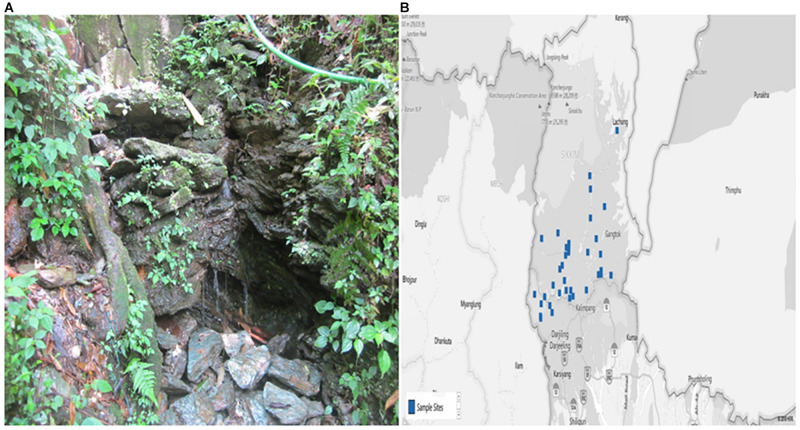
The photograph showing **(A)** Spring Water and **(B)** Map of the study site (blue dots specify the villages from where samples were collected) as previously reported (Singh et al., 2019). The map was collected from the Spring Detail (http://www.Sikkimsprings.org/dv/view.php) [Map chart was prepared in Microsoft Excel (office 365)] (Supplementary File: Table S1).

The study was conducted in different villages of Sikkim, which is a NorthEastern state of India. The State is located off the slopes of Eastern Himalayas between the latitude of 27° 05′ and 28° 07′ North and the longitudes of 87° 59′ and 88° 56′ East, covering approximately 115 km from North to South and 65 km from East to West. The landscape of this area varies with an altitude between 300 to 8583 m above sea level that comprises lower, middle and higher hills, alpine zones and snowbound land. Sikkim is a multi-ethnic state comprising of both tribal and non-tribal groups (Tambe et al., 2012). The Himalayan range is one of the important sources of numerous perennial rivers and springs (Tambe et al., 2012) (Figure 1).

### Study Design

For the current study, a total of 40 spring water samples (10 samples × 4 districts = 40 samples) were collected from four districts. Springs were selected based on the prevelance of waterborne diseases in a particular area with the help of the State Institute of Rural Development (SIRD), Government of Sikkim, India. The SIRD department helped in selecting the particular area of different districts, community, and blocks (village level blocks: VLB or Gram Panchyant Unit: GPU) which reported recent waterborne disease outbreaks. More than 80% of the springs water samples in that particular area were collected for microbiological and antibiotic resistance analysis. Springs were further selected based on the number of population dependent, altitude and perennial nature. Selection of perennial nature was based on the reason that those spring water will be used by the community throughout the year and large number of population will be depended. Higher the number of population dependency there will be higher risk of anthropoegenic contamination. Easy accessibility of springs at lower altitude could also be major contributing factor for water contamination (Supplementary File S1). The complete study design is summarized in Supplementary File S2.

### Sampling Procedure

Water samples were collected in 1L sterile, wide-mouth, plastic bottles (Nalgene, United States). Before collection, containers were sterilized by autoclaving and washed with aqueous sodium thiosulfate solution [100 g/l (w/v)]. While collecting the samples, bottles were completely submerged into the water and it was opened inside the water source to avoid air contamination. Containers were filled by holding it diagonally; lower part was submerged up to 30 cm with the mouth facing slightly upwards. A gap of 2 cm was left between the cap and water to provide sufficient airspace for the mixing of water before analysis (American Public Health Association, 1999).

### Enrichment of Water Sample

One milliliter of water samples were enriched in different enrichment media like Lauryol Tryptose Broth (Hi-media, M080), Gram-Negative Broth (Hi-media, N242), Selenite Cystine broth (Hi-media, M1079) and Alkaline Peptone Water (Hi-media, M618) and incubated at 37°C for overnight (American Public Health Association, 1999).

### Isolation of Bacteria

For isolation of bacteria, one loop full of enriched media was inoculated on different culture media like MacConkey and Eosin-Methylene Blue for isolation of *Escherichia coli, Klebsiella, Enterobacter, Hafnia, Morganella*, and *Citrobacter. Salmonella-Shigella* agar was used for the isolation of *Salmonella* and *Shigella* species. Thiosulfate Citrate Bile Salt agar was used for the isolation of *Vibrio* species (Murray et al., 2017).

### Identification of Bacteria

The isolates were identified by colony morphology, cell morphology and standard biochemical test as recommended in the Manual of Clinical Microbiology (Supplementary Files S3, S4) (Murray et al., 2017). Colony and cell morphology were summarized in Supplementary File S3. Gram staining method was used for cell morphology and different standard biochemical tests such as IMViC Test, different sugar fermentation test, amino acid metabolism tests were performed for identification of bacterial isolates (Supplementary File S4).

### Antibiotic Susceptibility Test

The antibiotic susceptibility test (AST) was performed using different classes of antibiotics *viz.* ampicillin (30 mcg, cat no. SD077, Hi-media), amoxicillin (30 mcg, cat no. SD0076, Hi-media), cefoxitin (30 mcg, cat no. SD041, Hi-media), ceftazidime (30 mcg, cat no. SD062, Hi-media), streptomycin (10 mcg, cat no. SD031, Hi-media), netillin (30 mcg, cat no. SD046, Hi-media), amikacin (30 mcg, cat no. SD035, Hi-media). Chloramphenicol (30 mcg, cat no. SD006, Hi-media), tetracycline (30 mcg, cat no. SD037, Hi-media), imipenem (10 mcg, cat no. SD073, Hi-media), ciprofloxacin (5 mcg, cat no. SD060, Hi-media), norfloxacin (10 mcg, cat no. SD057, Hi-media) and ofloxacin (5 mcg, cat no. SD087, Hi-media) by disk diffusion method. Selection of these antibiotics were made based on their differences in mode of action in bacterial cell. Ampicillin, cephalosporin, and imipenem target bacterial cell wall, streptomycin; tetracyclin inhibits protein synthesis and Quinolones inhibit bacterial DNA replication. A single colony of pure bacterial isolates was inoculated in 5 ml of sterile NaCl (0.9%) to make equivalent turbidity of McFarland 0.5 standard. After that, the suspension was evenly streaked on the Mueller-Hilton Agar (MHA) with a sterile cotton swab. The disks containing different concentrations of antibiotics were impregnated on the plate using sterile forceps (CLSI, 2014). The standard culture of *E. coli* MTCC10898 was used as an antibiotic sensitive control culture with each batch of AST.

The isolates which displayed resistance to three or more than three classes of antibiotics were designated as multi-drug resistant (MDR) bacteria. The Multiple Antibiotic Resistance (MAR) Index was calculated by using the formula (Krumperman, 1983):

$$\mathrm{MAR}=\frac{\mathrm{Number}\,\mathrm{of}\,\mathrm{antibiotics}\,\mathrm{to}\,\mathrm{which}\,\mathrm{an}\,\mathrm{isolate}\,\mathrm{showed}\,\mathrm{resistance}}{\mathrm{Total}\,\mathrm{no}\,\mathrm{of}\,\mathrm{antibiotics}\,\mathrm{used}}$$

### 16S rRNA Sequencing

Selected bacterial isolates were identified by 16S rRNA sequencing. Bacterial DNA was extracted by standard boiling lysis method. A single colony of each isolate were suspended in 100 μl of double distilled water and boiled for 10 min. After boiling, the samples were kept in ice for 10 min, followed by centrifugation for 5 min at 11,200 *g* to pellet down the cellular debris. Collected supernatant was used as a template for PCR amplification of 16S rRNA. A total of 25 μl reaction mixture was prepared with 2 μl of template DNA (100 ng/μl), 1 μl of universal 16S rRNA primers (27F 5′-AGAGTTTGATCMTGGCTCAG-3′ and 1492 R 5′-TACGGYTACCTTGTTACGACTT-3′) (10 Pico mole) (Woo et al., 2000), 12.5 μl of Go Taq Green Master Mix, 0.5 μl of 2X DNA polymerase (Promega, Madison, United States). The volume make-up was done with nuclease-free water. Reaction cycle used for amplification was as follows: initial denaturation, 95°C for 3 min; 30 cycle of denaturation at 95°C for 30 s, annealing at 54°C for 1 min, extension at 72°C for 1.30 min, final extension at 72°C for 10 min. Quality of the DNA was checked on 0.8% agarose gel and DNA was quantified using nanodrop at 260/280 ratio. The standard culture *E. coli* MTCC 1089 were used as a positive control for PCR amplification of 16S rRNA gene.

### Detection of Virulence Gene in *E. coli* by Multiplex PCR

DNA of *E. coli* isolates were extracted by the Phenol-Chloroform method as described by Sambrook and Russell (2002). All the multiplex PCR reactions were performed in 20 μl reaction mixture containing: template 0.5 μl, PCR master mix 10 μl containing Taq DNA polymerase 0.05U/μl, reaction buffer, 4 mM MgCl_2_, 0.4 mM of each dNTP (Thermo Fisher Scientific, cat. No: K0171) and 1 μl of each forward and reverse primer (Eurofins Scientific, India) (Table 1). The PCR reaction conditions were as follows: 95°C for 2 min, 95°C for 15 s, 52°C for 8 s and 72°C for 10 s, 30 cycles and a final extension at 72°C for 2 min. Amplified products were analyzed by 2% agarose gel electrophoresis with EtBr staining. Virulent STEC strain was selected for this study based on the fact that they can cause severe food borne and water borne diseases. Secondly, primary sources of STEC outbreaks are raw or undercooked ground meat products (Hemmatinezhad et al., 2015), raw milk (Momtaz et al., 2012), and fecal contamination of vegetables (Luna-Guevara et al., 2019; Reza et al., 2019) and it has been reported from Sikkim in previous studies (Bandyopadhyay et al., 2012).

**TABLE 1:** List of primers used for virulence gene detection, their sequence, and amplified product size.

Target Gene	Primers	Primer designation	PCR Product Size (bp)	References
*elt*	F 5′-ACGGCGTTACTATCCTCTC-3′	LT	273	Tobias and Vutukuru, 2012
	R 5′-TGGTCTCGGTCAGATATGTG -3′			
*stx1*	F 5′-CAGTTAATGTGGTGGCGAAGG-3′	Stx1	348	Tobias and Vutukuru, 2012
	R 5′-CACCAGACAATGTAACCGCTG-3′			
*eae*	F 5′-TCAATGCAGTTCCGTTATCAGTT-3′	Eae	482	Tobias and Vutukuru, 2012
	R 5′-GTAAAGTCCGTTACCCCAACCTG-3′			

### Detection of Antibiotic Resistance Genes in *E. coli*

Antibiotic resistance genes for tetracycline, streptomycin, quinolones, and ampicillin were detected using PCR (Table 2). A 25 μl PCR reaction mixture containing 12.5 μl 2X Thermo Fischer scientific master mix, 1 μl of 20 pmol of each primer i.e., forward and reverse primers (Table 2), double distilled water (8.5 μl) and DNA template (2 μl). PCR reaction conditions were as following: initial denaturation 95°C for 5 min, 35 cycles of 95°C for 30 s, 52°C for 1 min and 72°C for 1 min, with a final extension at 72°C for 7 min. Amplified products were analyzed by 1.5% agarose gel electrophoresis.

**TABLE 2:** List of ARGs primers, their sequence, and the amplified product size.

Target Gene	Primers	Primer designation	PCR Product Size (in bp)	References
Tetracycline	F 5′-AACTTAGGCATTCTGGCTCAC-3′	tetO	515	Al-Bahry et al., 2016
	R 5′-TCCCACTGTTCCATATCGTCA -3′			
Streptomycin	F 5′-TTTGCTGGTTACGGTGAC-3′	aadA1	497	Nahar and Rashid, 2018
	R 5′-GCTCCATTGCCCAGTCG-3′			
Quinolones	F 5′-GATCGTGAAAGCCAGAAAGG-3′	qnrB	469	Robicsek et al., 2006
	R 5′-ACGATGCCTGGTAGTTGTCC-3′			
Ampicillin	F 5′-TGGCCAGAACTGACAGGCAAA-3′	CITM	462	Amer et al., 2018
	R 5′-TTTCTCCTGAACGTGGCTGGC-3′			

### Statistical Analysis

Results were analyzed using Microsoft Excel (Office 365). The significance of the results were tested using *t*-test at *p* < 0.05 between presence of bacterial diversity, antibiotic resistance pattern, ARG and virulence gene of isolates from different spring water of Sikkim. Multivariate analysis like principle component analysis (PCA) was performed to establish correlation between the diversity of antibiotic resistant pathogens in different spring water of Sikkim using R statistics (Package:Factoextra). Correlation between antibiotic resistance and virulence gene was tested using Pearson-correlation test with R statistical package “PerformanceAnalytics.” One way ANOVA was used to see significance of the results and significant results were tested using *post hoc* Tukey’s honestly significant difference (HSD) for significant pairwaise comparison. After Tukey HSD, *p*-value was adjusted with Tukey correction factor. ANOVA and *post hoc* Tukey was analyzed using R statistics (package: rstatix and ggpubr).

## Results

### Microbial Diversity

**FIGURE 2:**
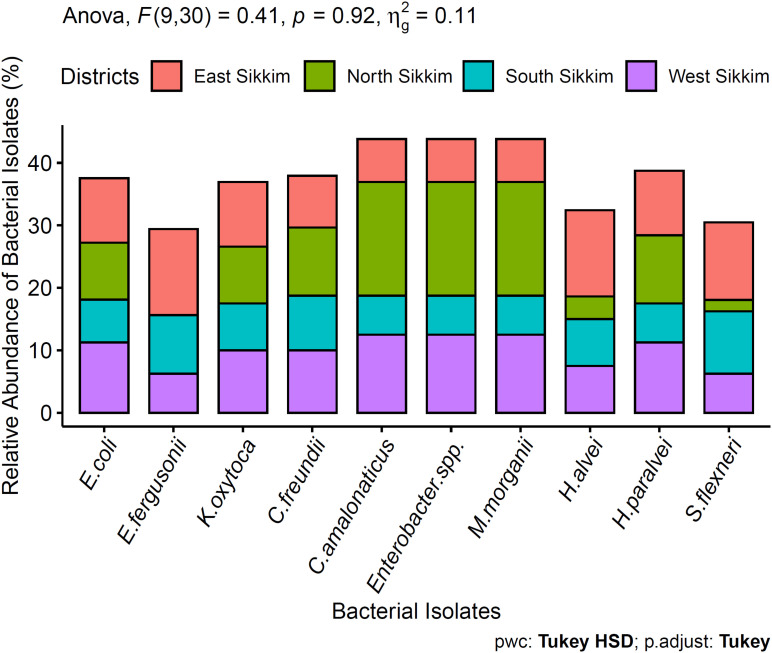
Dominant bacteria present in the spring water of Sikkim. ANOVA didn’t show any significant difference between the data.

**FIGURE 3:**
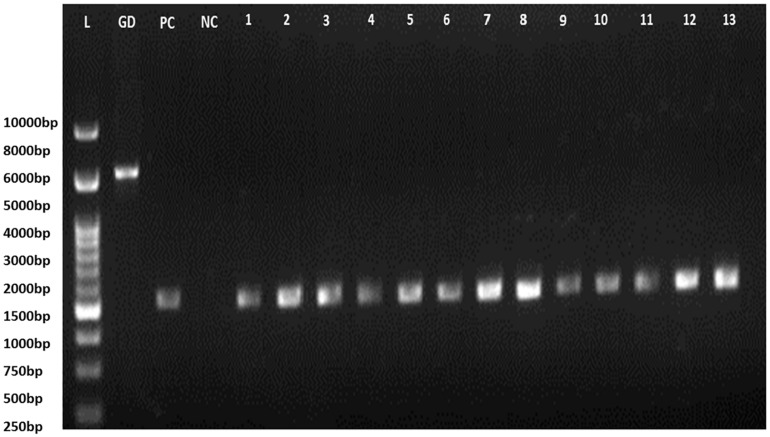
16S rRNA PCR gel electrophoresis (L, Ladder; GD, Genomic DNA; PC, positive control of *E. coli* amplified product; NC, Negative control; 1–13: isolates code).

Among the total 440 bacterial isolates, 10% of bacterial isolates were not representative of any clinically important pathogens and were not considered in this study. Out of 400 clinically importantbacterial isolates, a total of 36.36% were isolated from South Sikkim, 32.95% from East Sikkim, West Sikkim 18.18% and 12.50% from North Sikkim. All the spring water samples were positive for one or more *Enterobacteriaceae* isolates. The bacterial isolates were identified by colony morphology and cell morphology. All the 400 identified medically important bacteria (90%) were Gram-negative, rod-shaped bacteria, and belonged to *Enterobacteriaceae* family [90.90%, *p* < 0.05 (*p* = 0.03)]. Molecular characterization using 16S rRNA sequencing showed dominance of the phylum. The *Enterobacteriaceae* family members were predominant in all the water samples of Sikkim. Among the 400 clinically important bacteria isolated from different districts, *Citrobacter amalonaticus* [18.18%, *p* > 0.05, (*p* = 0.92)], *Enterobacter sp.* [18.18%, *p* > 0.05, (*p* = 0.92)], and *Morganella morganii* [18.18%, *p* > 0.05, (*p* = 0.92)] were predominant followed by *Escherichia fergusonii* [13.79%, *p* > 0.05, (*p* = 0.92)] and *Hafnia alvei* [13.79%, *p* > 0.05, (*p* = 0.92)]. District wise bacterial diversity showed that East Sikkim was mainly dominated by *Hafnia alvei* [13.79, *p* > 0.05, (*p* = 0.92)] and *Escherichia fergusonii* [13.79, *p* > 0.05, (*p* = 0.0001)] whereas South Sikkim was dominated by *Shigella flexneri* [10%, *p* < 0.05, (*p* = 0.92)]. The *Citrobacter amalonaticus* [12.5%, *p* > 0.05, (*p* = 0.92)], *Enterobacter* spp. [12.5%, *p* > 0.05, (*p* = 0.92)] and *Morganella morganii* [12.5%, *p* > 0.05, (*p* = 0.92)] were dominant in the West Sikkim whereas North Sikkim was dominated by *Citrobacter amalonaticus* [18.18%, *p* > 0.05, (*p* = 0.92)], *Enterobacter* spp. [18.18%, *p* > 0.05, (*p* = 0.92)] and *Morganella morganii* [18.18%, *p* > 0.05, (*p* = 0.92)] (Figure 2). All the 16S rRNA sequences were submitted to NCBI and accession number were summarized in Supplementary File S5 and Figure 3.

### Antibiotic Resistance in the Bacterial Isolates of Spring Water of Sikkim

**FIGURE 4:**
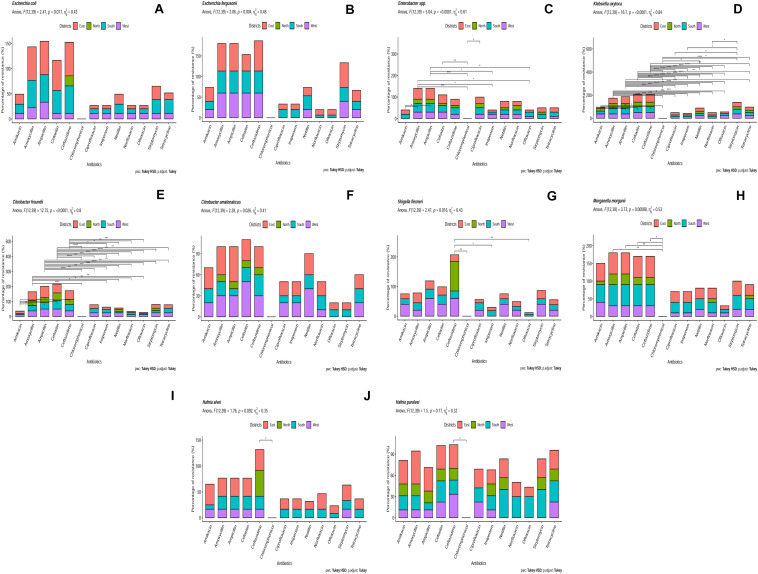
Antibiotic resistance pattern in **(A)** *Escherichia coli*, **(B)** *Escherichia fergusonii*, **(C)** *Enterobacter sp.*, **(D)** *Klebsiella oxytoca*, **(E)** *Citrobacter freundii*, **(F)** *Citrobacter amalonaticus*, **(G)** *Shigella flexneri*, **(H)** *Morganella morganii*, **(I)** *Hafnia alvei*, **(J)** *Hafnia paralvei* (showed statistical significance at *p*-value ^∗^*p* < 0.05; ^∗∗^*p* < 0.01; ^∗∗∗^*p* < 0.001; ^****^p < 0.0001).

Identified bacterial isolates were further subjected to AST (Supplementary File S6). *Citrobacter freundii* isolates were highly resistant to ampicillin (75%). *Escherichia fergusonii* isolates were resistant to ceftazidime (73.30%). *Escherichia coli* isolates were resistant against both ampicillin (66.66%) and amoxicillin (66.66%) and *Klebsiella oxytoca* isolates were resistant against cefoxitin (66.66%). The least resistance was found against ofloxacin (5.55%) by *Shigella flexneri* (Figure 4). Antibiotic resistance was recorded in following order (decreasing order): ampicillin, amoxicillin, cefoxitin, ceftazidime, streptomycin, amikacin, tetracycline, netillin, ciprofloxacin, norfloxacin, imipenem and ofloxacin. Bacterial isolates from East Sikkim showed resistance against highest number of antibiotics followed by South and West Sikkim whereas the least resistance was detected in bacterial isolates from North Sikkim. Several MDR strains were also detected. *Citrobacter amalonaticus*, which showed resistance to ampicillin (50%), cefoxitin (50%), netillin (40%), amoxicillin (40%). *Enterobacter* sp. showed resistance to ampicillin (50%), amoxicillin (50%), and cefoxitin (40%). *Morganella morganii* showed resistance to ampicillin, amoxicillin, cefoxitin, and ceftazidime with each accounting for 60% (Figure 4). *Hafnia alvei* isolates showed resistance to ceftazidime (50%), amikacin (40%), ampicillin (35%), amoxicillin (35%), and cefoxitin (35%) (Figure 4). However, all isolates showed 0% resistance to chloramphenicol (Figure 4). *Shigella flexneri, E. coli* and *Hafnia alvei* isolates from the North Sikkim showed resistance to ceftazidime only (100, 20, 50%) (Figure 4).

### Multiple Antibiotic Resistance Index

**FIGURE 5:**
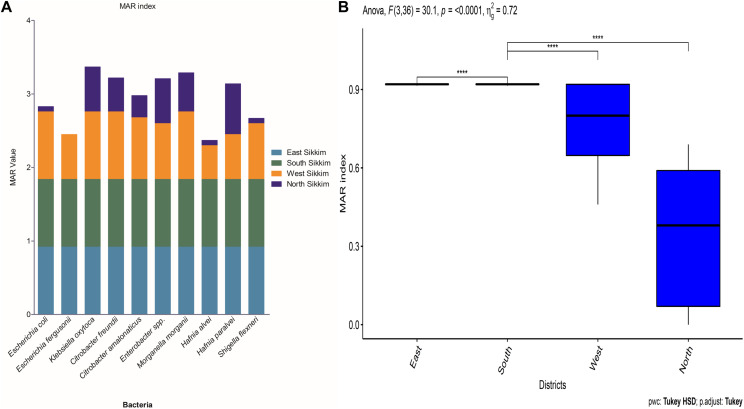
**(A)** The graph showing the MAR index of bacterial species from different districts of Sikkim. **(B)** Statistical significance between the data of different districts (^∗^showed statistical significance at *p*-value ^****^p < 0.0001).

Multiple antibiotic resistance (MAR) indexing is one the easiest method for making distinction in antibiotic resistance pattern among different isolates and for making health risk assessment. It indicates whether the identified isolates are from a region of high or low antibiotic use. The MAR index value greater than 0.2 indicates higher risk of source contamination where antibiotics are most often used (Davis and Brown, 2016). MAR value equals to 1 indicates 100% resistance of the isolate to the tested antibiotics and the source is at higher risk due to frequent use of antibiotics. East and South Sikkim bacterial isolates showed MAR index of 0.92 whereas the MAR index of West Sikkim isolates ranged from 0.46 to 0.92. Isolates from North Sikkim showed the lowest MAR index (0 – 0.61) (Figure 5A). Test of analysis of variance showed significant difference among the mean of the results, and the *post hoc* Tukey showed significant difference among east, west, south, and north districts data (Figure 5B).

### Principal Component Analysis Based on Antibiotic Resistance

The correlation between spring water of different districts of Sikkim using antibiotic resistant isolates as an exploratory variable was carried out by principal component analysis (PCA). The F1 component (principal component 1) possessed 67.40% variability whereas F2 (principal component 2) showed 20.50% variability and together they accounted for 87.90% variability of the data. Correlation between the water samples of different districts of Sikkim was significant with a *p*-value < 0.05. A significant positive correlation was observed between East and South Sikkim water samples. Water samples from West and North Sikkim were totally different in terms of distribution of antibiotic resistant isolates.

### Detection of Virulence Gene in *E. coli*

**FIGURE 6:**
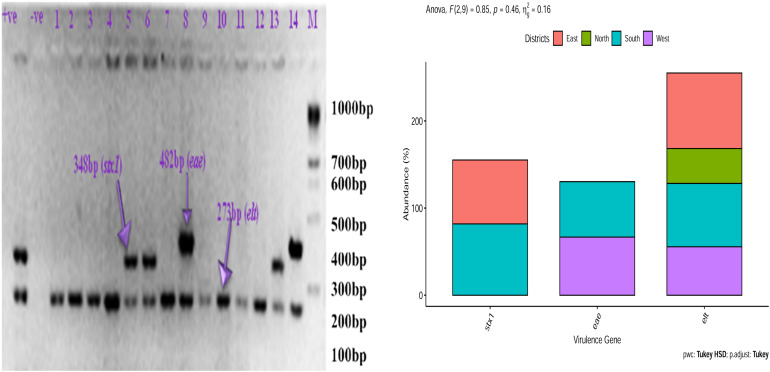
Prevalence of virulence gene in *Escherichia coli* isolated from spring water of different districts of Sikkim (+ve, positive control; –ve, negative control).

*Escherichia coli* isolates from East Sikkim carried the lowest percentage of *stx1* (73.33%, *p* < 0.05) gene and highest percentage of *elt* gene (86.66%, *p* < 0.05). *E. coli* isolates from North Sikkim showed single virulence gene *viz. elt* (40%, *p* < 0.05). Isolates from West Sikkim carried highest percentage of *eae* (66.66%, *p* < 0.0004) gene followed by *elt* gene (55.55%, *p* < 0.0001). *E. coli* isolates from South Sikkim showed the highest percentage of *stx1* (81.81%, *p* < 0.0001) gene followed by *elt* (72.72%, *p* < 0.0001) and *eae* (63.63%, *p* < 0.0004) gene (Figure 6).

### Detection of Antibiotic Resistance Gene in *E. coli* Isolates of Spring Water of Sikkim

**FIGURE 7:**
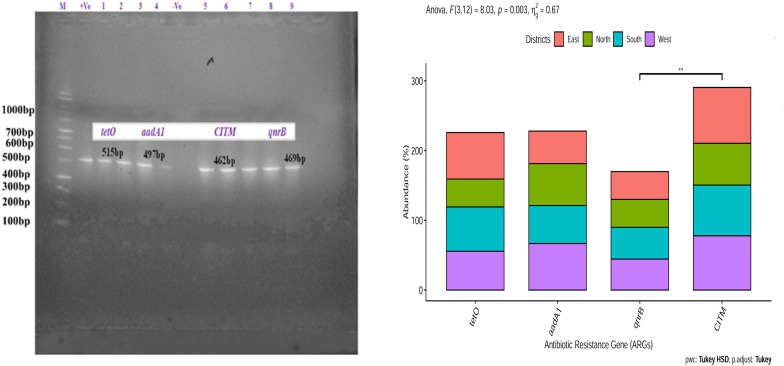
Prevalence of antibiotic resistance gene in *Escherichia coli* isolates from spring water of different districts of Sikkim. ^∗^Showed statistical significance at *p* value ^∗∗^*p* < 0.01.

*Escherichia coli* isolates from East and South Sikkim had the highest percentage of ampicillin (*CITM*) (80%, *p* < 0.05, *p* = 0.0079) resistance gene followed by that of tetracycline (*tetO*) (66.66%, *p* < 0.05, *p* = 0.0079) and quinolones (*qnrB*) (40%, *p* < 0.05, *p* = 0.0079). *E. coli* isolates from West Sikkim showed highest percentage of ampicillin (77.77%, *p* < 0.05, *p* = 0.0034) resistance gene followed by that of streptomycin (66.66, *p* < 0.05, *p* = 0.0034) and quinolones (44.44%, *p* < 0.05, *p* = 0.0034). *E. coli* isolates from North Sikkim showed highest percentage of ampicillin (60%, *p* < 0.05, *p* = 0.0032) and tetracycline (60%, *p* < 0.05, *p* = 0.0032) resistance genes followed by that of streptomycin (40%, *p* < 0.05, *p* = 0.0032) and quinolones (40%, *p* < 0.05, *p* = 0.0032) (Figure 7).

### Correlation Between Antibiotic Resistance and Virulence Gene

**FIGURE 8:**
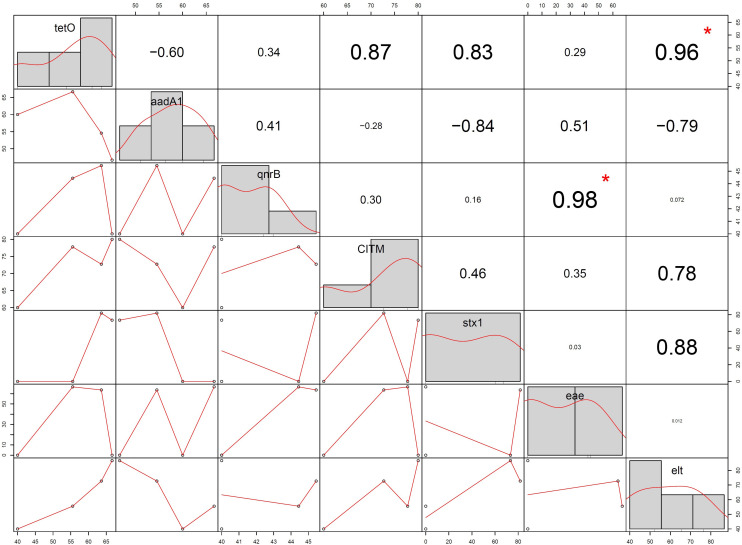
Correlation between antibiotic resistance gene and virulence gene of *Escherichia coli* isolated from spring water of Sikkim (+ve, positive control; –ve, negative control; (^∗^ = statistically significant).

In Pearson correlation between ARG and virulence gene of *E. coli*, it was found that tetracyclin resistance (*tetO*) gene showed positive association (0.96) with *elt* gene (statistically significant at *p* < 0.05) and *stx1* gene (0.83) (non-significant). Whereas quinolones (*qnrB*) resistance gene showed positive correlation (0.98) with *eae* gene with statistical significance (*P* < 0.05). Streptomycin resistance gene (*aadA1*) showed negative correlation with *stx1* (−0.84) and *elt* (−0.78) gene whereas ampicillin resistance gene (*CITM*) showed positive correlation (0.78) with *elt* gene but statistically non significant (Figure 8).

## Discussion

Poor hygiene and sanitation, and the contaminated water accounts for 6.3% of global deaths and 9.1% of the total global disease burden (Pal et al., 2018). In developing countries, water contamination is very common due to lack of proper water treatment systems and has become a reason for deaths of several hundred to thousands each year (Pruss-Ustun et al., 2008; Bain et al., 2014). Contamination of water sources is a concerning issue around the world because of the increasing population, climate change and anthropoegenic activities. Water quality defines health quality of a community in a population. Identification of contaminated water sources especially in developing countries and rural areas has become a need of the moment to manage and minimize the future health risks. In the Eastern Himalayan state Sikkim, where water sources are limited and altitude variation restricts the people to make use of mountain springs as their sole source of potable water. However, concerning question is, majority of the Sikkimese people do not use any form of filtration or pretreatment for using the spring water as a potable source. Using untreated spring water as a potable source increases the risk of transmission and getting in contact with water borne pathogens. Our previous study (Singh et al., 2019) documented how spring water and community reservoir (the place where the spring water is collected/stored prior to its supply to households) of the state can seed the future health risks owing to its alarming fecal contaminations.

Pharmaceuticals have improved the quality of life and public health, especially pharmaceutical products like antibiotics have acted as a wonder medicine that saved countless lives (Davies and Davies, 2010). However, excessive use of antibiotics in agriculture, animal husbandry, animals and humans worldwide plays a very important role in the emergence of ARB (Singh et al., 2018a; Almakki et al., 2019). Overuse of antibiotics has increased the accumulation of active antimicrobial compounds in the environment and favored the selection of ARGs and ARB (Almakki et al., 2019). Surface water remains a connecting link between the emergence and distribution of antibiotic resistance among pathogens due to direct disposal of fecal and agricultural runoff, and the hospital and municipal wastewater effluents (Grenni et al., 2018). Once the antibiotic resistance bacteria enters the waterways, they can either be consumed by humans or animals and can cause infections, or they can transfer ARGs to other bacteria which are present in that environment (Sanderson et al., 2018). In Sikkim, to maintain the continuity of water supply to the community, spring water is being collected and supplied to the community via pipelines. There have been several recent outbreaks of waterborne diseases in different areas of the state which has raised the concern about water quality. A recent report from the Ministry of Health and Family Welfare, Government of India, indicated that cholera, acute diarrheal diseases and enteric or typhoid fever is becoming a health risk in the state which needs early attention (Government of India, Ministry of Health & Family Welfare, 2019). A total of 41816 cases of acute diarrheal disease were reported in 2017 and 41449 cases in 2018. A total of 104 cases of enteric fever were reported in 2017, and 158 cases in 2018 by the Sikkim state authorities. These numbers are alarming and our previous study also reported severe fecal contamination in the spring water of Sikkim, which needs immediate attention (Singh et al., 2019).

In this study we tried to characterize the distribution of ARB belonging to *Enterobacteriaceae* family and occurrence of ARGs and VGs in isolated *E. coli* from potable spring water sources. Characterization of *Enterobacteriaceae* ARBs from spring water showed a dominance of *Escherichia coli, E. fergusonii, Citrobacter freundi, C. amalonaticus, Hafnia alvei, Morganella morganii, Enterobacter* sp. and *Shigella flexneri* (Figure 2). Antibiotic susceptibility study showed that *E. coli* isolates were resistant to ceftazidime (73.33%), ampicillin (66.66%) and amoxicillin (66.66%). However certain degree of resistance of the *E. coli* isolates was also observed against norfloxacin (6.66%) and ofloxacin (6.66%). *E. coli* isolates were also found resistant to tetracycline (26.66%) and streptomycin (60%) (Figure 4A). The resistance of *E. coli* to common antibiotics is becoming a serious concern for clinical facilities. Reports of multiple antibiotic-resistant *E. coli* isolates from water sources can be found worldwide. A study from Central Michigan had reported *E. coli* isolates from water samples, human septage, domestic fecal matter, and wild animal fecal matter to be resistant to tetracycline (27.30%), cephalothin (22.70%), sulfisoxazole (13.30%), and streptomycin (13.10%) (Sayah et al., 2005). Another study from Leaon, Nicaragua showed widespread resistance to Ampicillin (100%), chloramphenicol (69%), ciprofloxacin (69%), and nalidixic acid (70%) in *E. coli* isolated from well water, sewage water, and hospital effluents (Amaya et al., 2012). On an intriguing and contrary note *E. coli* isolates from spring water of Sikkim did not showed any resistance to chloramphenicol. The probable reason behind the chloramphenicol susceptibility of the isolates may be the result of infrequent consumption of chloramphenicol in the region as this antibiotic is prescribed for diseases like typhoid, cholera, conjunctivitis, etc. which are less prevalent in Sikkim (Singh et al., 2018c). The possible reason behind the development of resistance to the common antibiotics could be the frequent use or misuse of antibiotics due to easy availability and affordability (Shakya et al., 2013). In India, *E. coli* isolated from the Ganga river showed resistance to antibiotics cephalosporin (37%), ampicillin (20%), amoxicillin (20%), tetracycline (20%), and ciprofloxacin (5.30%) (Biswas et al., 2015). Similarly, several studies had reported the presence of antibiotic-resistant *E. coli* in different environmental and clinical samples (Sayah et al., 2005; Ram et al., 2007, 2008; Li et al., 2010; Tagoe et al., 2011; Guo et al., 2014, 2017; Adzitey et al., 2015; Chen et al., 2017; Muringani, 2017; Amer et al., 2018; Diwan et al., 2018; Igwaran et al., 2018; Lu et al., 2018; Mishra et al., 2018; Mitali et al., 2018; Vital et al., 2018; Amador et al., 2019; Sandhu et al., 2019). Study on distribution of ARGs in the present study reflects that the spring water of Sikkim are reservoirs of multiple ARGs. Isolated *E. coli* from spring water of Sikkim showed the presence of tetracycline (*tetO*), streptomycin (*aadA1*), quinolones (*qnrA*) and ampicillin (*CITM*) resistance genes (Figure 7). In a similar study, *E. coli* isolated from river Ganga were found to have genes for neomycin (100%), streptomycin (33.30%), and ampicillin (6.60%) resistance (Ram et al., 2007). Different surface water samples from Hangzhou city, China had reported tetracycline resistance gene (89.02%) in *E. coli* isolates (Chen et al., 2017). The occurrence of plasmid-encoded tetracycline resistance gene among *E. coli* in the spring water of Sikkim may increase the possibility of horizontal gene transfer in susceptible bacteria. ARG in the *E. coli* isolated from foods, and clinical and environmental samples had been frequently reported in many developing countries (Ram et al., 2007, 2008; Li et al., 2010; Guo et al., 2014, 2017; Chen et al., 2017; Amer et al., 2018; Diwan et al., 2018; Sandhu et al., 2019).

Humans and animals both are colonized by different strains of *E. coli* but their colonization is asymptomatic while expression of *stx, elt*, or *eae* genes defines the pathogenesis in humans (Ram et al., 2007). *E. coli* isolated from spring water of Sikkim showed highest percentage of *elt* gene (63.73%) followed by *stx1* gene (38.78%) and *eae* gene (32.57%) (Figure 6). The presence of *stx1* gene in *E. coli* showed the prevalence of STEC (Shiga toxin-producing *E. coli*) in the spring water of Sikkim and indicated animal or human origin fecal contamination of water bodies. A similar study on *E. coli* isolates from river Ganga and Gomati, India showed the presence of virulence genes such as *stx1* (82%), *stx2* (82%), *eae* gene (70.60%), and *elt* gene (33.30%) (Ram et al., 2007, 2008). Due to a very low infection dose (1-10 colony-forming unit) with short incubation period (3 h) Shiga toxin-producing *E. coli* (STEC) have a very high rate of transmission and infection to humans and animals (Kuhnert et al., 2000; Ranjbar et al., 2017). Drinking water contaminated with STEC can cause diarrheal disease in humans and animals (Obi et al., 2004). Several studies reported the presence of virulence gene *stx* in *E. coli* from human stool samples, environmental samples, fermented dairy products and food samples (Kuhnert et al., 2000; Qadri et al., 2005; Ram et al., 2007, 2008; Sidhu et al., 2013; Dehkordi et al., 2014; Ranjbar et al., 2017, 2018). Expression of *eae* gene is essential for the complete functioning of virulence gene STEC (*stx1* and *stx2*) in humans. The expression of *stx1* and *stx2* gene leads to hemorrhagic colitis (HC) and hemolytic uremic syndrome (HUS) (Obi et al., 2004). The strain of *E. coli* which bear heat-labile (*LT*) and heat-stable (*ST*) enterotoxin along with colonizing factor were described as ETEC strain (Gaastra and Svennerhoim, 1996), and 63.73% of *E. coli* isolates from the spring water of Sikkim had *elt* gene. Similar observations have been made in the surface water studies conducted in India, Bangladesh and South Africa (Gaastra and Svennerhoim, 1996; Kuhnert et al., 2000; Obi et al., 2004; Ram et al., 2007). Gain and loss of VGs by *E. coli* can determine the fate of pathogenesis (Ram et al., 2007). Thus, the presence of different plasmid-encoded virulence genes (*elt, stx1, eae*) bearing *E. coli* strain in the spring water of Sikkim indicates possible future health risks. Poor sanitation facility and population pressure have increased the probability of the presence of virulent gene harboring pathogenic bacteria in the surface water of developing countries.

*Escherichia fergusonii* (7.35%) was reported from the spring water of Sikkim (Figure 2). *Escherichia fergusonii* are rarely occurring, emerging pathogens for humans and animals. It was earlier known as enteric group 10 and named after American microbiologist William H. Ferguson (Farmer et al., 1985). *Escherichia fergusonii* isolates from spring water of Sikkim showed resistance to different classes of antibiotics including ceftazidime (73.33%), streptomycin (60%), ampicillin (60%), and cefoxitin (40%) (Figure 4B). A similar study from Turkey found *E. fergusonii* isolates were resistant to penicillin G (100%), erythromycin (76.90%), and oxytetracycline (23%) (Parin et al., 2018). MDR *E. fergusonii* was also isolated from the Orissa (Mahapatra et al., 2009).

Another major pathogenic bacterium identified from the spring water of Sikkim was *Klebsiella.* It is the second leading cause of nosocomial infection after *E. coli* among *Enterobacteriaceae*. *Klebsiella* are opportunistic pathogens that cause several diseases like pneumonia, urinary tract infection (UTI), soft tissue infection, and septicemia (Hagiwara et al., 2013). Taxonomic identification showed 9.23% of *Klebsiella* isolates from spring water were *K. oxytoca* (Figure 2). However, a patient infected with *K. oxytoca* may remain asymptomatic but it is still considered as an opportunistic pathogen associated with nosocomial infection in cohorts of hospitalized patients including neonates and children (Savino et al., 2009, 2011; Gajul et al., 2015; Singh et al., 2016). Recent studies showed that *Klebsiella oxytoca* is becoming an emerging threat with higher acquisition of antibiotic resistance to common antibiotics than by other major pathogens of genus *Klebsiella viz., Klebsiella pneumoniae* (Singh et al., 2016). *K. oxytoca* isolates from spring water of Sikkim showed resistance to cefoxitin (66.66%), ceftazidime (66.66%), ampicillin (60%), and streptomycin (40%) (Figure 4D). Singh et al. (2016) from India, reported *K. oxytoca* from different clinical samples that showed resistance to ceftriaxone (72%), imipenem (59%), amikacin (71%), gentamicin (71%), ciprofloxacin (81%), and piperacillin/tazobactam (75%). *K. oxytoca* isolated from urine samples of UTI infected Turkish children showed resistance to ampicillin (97%), cephalothin (48.40%), cefuroxime (36.40%), amoxicillin (18.20%), and ceftriaxone (7.10%) (Gunduz and Uludað Altun, 2018).

*Citrobacter spp.* are commensal bacteria present in intestinal tracts of humans and several animals (Liu et al., 2017). *Citrobacter spp.* has been also isolated from water, soil and sewage (Lee et al., 2010; Liu et al., 2017). *C. freundii* (9.48%) and *C. amalonaticus* (10.95%) were the two major species isolated from the spring water of Sikkim (Figure 2). AST of *C. freundii* showed its resistance to ampicillin (75%), amoxicillin (58.33%), cefoxitin (58.33%), tetracycline (25%), and streptomycin (25%). Whereas *C. amalonaticus* showed resistance to ampicillin (50%), cefoxitin (50%), amoxicillin (40%), and netillin (40%) (Figures 4E,F). Commensal *C. freundii* isolated from the stool samples of children aged 3–14 years showed resistance to ampicillin (100%), Co-trimoxazole (100%), cefotaxime (100%), amoxiclav (100%), ciprofloxacin (100%), and imipenem (100%) whereas no resistance was reported against gentamicin and amikacin (Sandhu et al., 2019). *C. freundii* isolated from different clinical samples such as urine, stool, blood, and pus from India showed resistance to norfloxacin (79.59%), ciprofloxacin (71.53%), ceftazidime (69.39%), cefotaxime (67.35%), and meropenem (4.08%) (Khanna et al., 2012).

*Enterobacter* is a genus of Gram-negative, rod-shaped, facultative anaerobe, non-spore forming bacteria belonging to family *Enterobacteriaceae.* They are associated with bacteremia, septic arthritis, endocarditis, urinary tract infection, respiratory tract infection, intra-abdominal infection, skin and soft tissue infection (Singh et al., 2018c). *Enterobacter* spp. isolated from the spring water of Sikkim (Figure 2) showed resistance to ampicillin (50%), cefoxitin (40%), netillin (30%), ciprofloxacin (30%), tetracycline (30%), and imipenem (10%) (Figure 4C). *Enterobacter spp.* isolated from pharmaceuticals wastewater of Southwestern Nigeria showed resistance to ampicillin (100%), amoxicillin (100%), chloramphenicol (100%), sulfonamide (100%), trimethoprim (98%), and streptomycin (92%) (Obayiuwana et al., 2018). However, *Enterobacter* spp. isolates from the spring water of Sikkim did not show any resistance to chloramphenicol. The majority of the studies, has indicated the widespread multidrug resistance in *Enterobacter* spp. isolates against ampicillin, cephalothin, cefoxitin, septrin, chloramphenicol, amoxicillin, streptomycin, and ciprofloxacin (Bolaji et al., 2011; Jabbar Ibrahim and Kareem Hameed, 2015). In the United States, *Enterobacter* spp. isolates are the second most commonly encountered carbapenem resistant *Enterobacteriaceae* members which had progressively contributed to the spread of the carbapenem-resistant infection (Annavajhala et al., 2019). Multi-drug resistant *Enterobacter* spp. (13.44%) were isolated from the Cauvery river, India, in which about 93.85% isolates showed full resistance to all the 32 antibiotics used in that study (Skariyachan et al., 2015)

*Morganella* is a genus of Gram-negative, rod-shaped, facultative anaerobic bacteria, first isolated from pediatric fecal culture by Morgan et al. (1906). This bacterium is normally found in the environment and intestinal tract of mammals as a part of normal flora (Liu et al., 2016). *Morganella morganii* acts as opportunistic pathogen which causes sepsis, abscess, bacteremia, and UTI (Hawkey et al., 1983; Liu et al., 2016; Simkhada et al., 2016; Ibrahim et al., 2017; Guo et al., 2019; Minnullina et al., 2019). Isolates of *Morganella morganii* (10.95%) from spring water of Sikkim (Figure 2) showed resistance to ampicillin (60%), cefoxitin (60%), amikacin (50%), streptomycin (40%), and tetracycline (30%) (Figure 4H). *Morganella morganii* is reported from many UTI patients (Liu et al., 2016; Hawkey et al., 2018; Minnullina et al., 2019). *Morganella morganii* isolated from various hospital samples in China showed resistance to tetracycline, amikacin, gentamicin, ceftazidime, cefepime, imipenem, meropenem (Guo et al., 2019). Guo et al first time reported the NDM-5 producing *Morganella morganii* (Guo et al., 2019). From a tertiary care hospital of Nepal (a Himalayan country), *Morganella morganii* showed resistance to chloramphenicol (100%), cefixime (100%), cefotaxime (100%), gentamicin (100%), amikacin (100%), and imipenem (100%) (Simkhada et al., 2016). However, *Morganella morganii* isolates from Sikkim did not show any resistance to chloramphenicol. Multidrug resistant *Morganella morganii* (5.59%) was reported from Nigerian hospital wastes (Mustapha and Imir, 2019). Cephalosporin (0.50%) and ciprofloxacin (0.50%) resistant *Morganella morganii* were reported from a healthy community of Chandigarh, India (0.50%) (Monica et al., 2018).

*Hafnia alvei* (8.10%) and *H. paralvei* (9.68%) were identified from the spring water of Sikkim (Figure 2). *Hafnia alvei* showed resistance to ceftazidime (40%), amikacin (40%), ampicillin (35%), and cefoxitin (35%) and *Hafnia paralvie* were found resistant to amoxicillin (46.66%), streptomycin (40%), netillin (40%), and cefoxitin (33.33%) (Figures 4I,J). *Hafnia alvei* is a Gram-negative, rod-shaped, gastrointestinal commensal bacteria belonging to family *Enterobacteriaceae* and is an opportunistic pathogen. They cause nosocomial infection, bacteremia, peritonitis, UTI, and pneumonia (Janda and Abbott, 2006; Lancelevee et al., 2014; Loya and Walsh, 2015; Litrenta and Oetgen, 2017; Baral et al., 2018). In a study from Nepal, *Hafnia alvei* was isolated and identified from an 11 months old child who suffered from bacteremia following bronchopneumonia (Baral et al., 2018). From different clinical samples of India, such as urine, wounds, blood, cerebrospinal fluid (CSF), etc. extensively resistant and pan drug-resistant *H. alvei* had been reported (Bhatt et al., 2015).

*Shigella* a genus of Gram-negative, rod-shaped, entero-invasive bacteria causes shigellosis which is responsible for significant mortality and morbidity in young children and immunocompromised adults (Ranganathan et al., 2019). In the developing world *S. flexneri*, while in the more-industrialized regions *Shigella sonnei*, are responsible for the majority of disease burden (Kahsay and Muthupandian, 2016). Shigellosis is a global human health problem that kills about 700,000 peoples each year worldwide of which most belong to the age group of below 5 years (DuPont et al., 1989). *Shigella flexneri* was the dominant species identified from the spring water of Sikkim (Figure 2). East Sikkim (12.41%) being the hub of industrial development of the state counted the highest number of *Shigella flexneri* followed by South (10%) and North Sikkim (1.82%). *Shigella flexneri* isolates showed resistance to ceftazidime (60%), cefoxitin (40%), streptomycin (40%), netillin (40%), amikacin (40%), and ofloxacin (5.50%). *S. flexneri* isolates from North Sikkim showed resistance to only ceftazidime (100%) (Figure 4G). Similar antibiotic resistance profile can be found in the *Shigella flexneri* (71.1%) isolates from Shanxi Province, China. Isolates were resistant to ampicillin (98.50%), ticarcillin (95%), trimethoprim/sulfamethoxazole (85.20%), and chloramphenicol (78.60%) (Wang et al., 2019). In another study, *Shigella flexneri* (21%) isolates from Iran were found to show resistance to cotrimoxazole (100%), ampicillin (100%), tetracycline (75%), chloramphenicol (50%), cefixime (50%), and ampicillin (33.30%) (Abbasi et al., 2019). A study from Kerala, India reported *Shigella flexneri* isolates to display resistance to nalidixic acid (88.80%), ampicillin (83.30%), cotrimoxazole (83.30%), ciprofloxacin (77.70%), and furazolidone (38.80%) (Madhavan et al., 2018). The Oceanic region of Australia had also reported ampicillin (77%), tetracycline (74%), chloramphenicol (60%), and sulfamethoxazole/trimethoprim (49%) resistant *Shigella flexneri* (Malau et al., 2018). The children from Southern Mozambique had been recently shown to carry *Shigella flexneri* (70.1%) which were resistant to sulfamethoxazole/trimethoprim (62.70%), tetracycline (49.30%), ampicillin (46.30%), and chloramphenicol (44.80%) (Vubil et al., 2018). However, no chloramphenicol resistance was observed by us in the isolates from Sikkim.

Though a large number of antibiotics were used in the current study to detect the prevalent antibiotics resistance pattern, our study was limited by the use of bacteria belonging to *Enterobacteriaceae* family for assessing the spread of antibiotics resistance. So, there exists a chance that more resistant bacteria from different families might have been missed out, and the estimated antibiotics resistance rate might be an underestimation of the problem. However, lack of any water quality studies especially on antibiotic resistance pattern and distribution of antibiotic resistant pathogen makes it unique and it provides a baseline for future water quality research. This study suggested that spring water of Sikkim have high antibiotic resistance bacteria and ARG. Several risk factors such as direct antibiotic application, antibiotic resistance feed as well as environmental factors may have played vital role in distribution of antibiotic resistance in this ecosystem. More studies are needed to reveal a broad picture of antibiotic resistance in spring water of Sikkim and to set standard protocols or policies for proper treatment and management of spring water. Springs are free flowing ground water which flows in the form of surface water. So, there might be chances of contamination either at source, during free-flowing or during distribution of the water up to the household level. Some of the basic ground level management protocols which can be helpful in maintaining the water quality are:

(1) Springs should be protected from unnecessary anthropogenic/human disturbances.
(2) Frequent cleaning of the community water tanks and supply system using standard protocols.
(3) Prevent mixing of runoff water during rainy season to spring water, which carries large number of waste material including fecal from animal and household discharges.
(4) Proper chlorination of water source before supplying to the household.
(5) Water samples must be tested for both physicochemical and microbiological quality at frequent intervals (e.g., monthly, quarterly). Before supplying to the households, quality indicator/parameters should be checked and compared with standard guidelines of BSI and WHO.
(6) Putting fences around the springs to prevent direct access to animals as well as humans.
(7) Periodical inspection for the presence of indicator organism (fecal coliforms) in the spring water before supply (e.g., monthly, quarterly).
(8) Prevent disposal of any kind of waste material near the spring water sources.
(9) At household level, proper boiling or filtration before consumption is recommended.
The community needs to be educated about general hygiene, different methods available for household-level water treatment, and the importance of clean water for children and their health to avoid impending health disaster associated with the unsafe water supply.

## Conclusion

Antibiotic resistance has become a major challenge worldwide, especially in developing countries like India. Easy accessibility of antibiotics, self-prescription, incomplete treatment course of antibiotics combined with poor management of waste disposal, poor water quality and hygiene contribute to rapid distribution and occurrence of antibiotic resistance in pathogenic and environmental bacteria. The present study showed there is a high incidence rate of multiple antibiotic resistant enteric bacteria in the spring water of Sikkim and suggests spring water are not suitable for drinking without prior treatment or filtration. The Sikkimese community may be at greater risk of contracting infection due to extensive use of spring water for drinking, cooking and other household purposes. The high rate of antibiotic resistance occurrence among environmental bacteria could be a result of indiscriminate use of antibiotics in human, animal and agricultural sectors. The presence of MDR enteric Gram-negative bacteria in the spring water of Sikkim warrants an instant commitment to the rational use of antibiotics as much as possible. The study indicates the emerging imminent threat from antibiotic-resistant pathogens in the area and mandates for immediate remedial measures including educating the community about proper sanitation, hand hygiene, and drinking water treatment practices to successfully contain the impact on the community. The extent of the problem of this emergence of antibiotic resistance can be managed by conducting antibiotic surveillance program, sensible use of antibiotics, use of the alternative bio-control agents, creating awareness in public about the rational use of antibiotics, and intensive effort between students, doctors, research scientist, industrial participants and policymakers. This study also warrants further examination of spring water samples from surrounding area of South East Asia that share similar geography and have similar livelihood practices to plan a comprehensive approach for the containment of the emerging health problem in the region.

## Dedication Note From Samer Singh

This paper is dedicated to the fond memory of Dr. Hare Krishna Tiwari – a great friend, colleague, and collaborator. His sudden passing away on 27th September 2020, when this article was in production, deeply stunned and saddened us and so many other colleagues alike in the field. His scholarly pursuit, perseverance, and dedication to the field as well as the mentoring and career counseling of scholars will be passionately remembered. This article will bring back the fond memories of what a pleasure it was to be his friend, colleague, and collaborator. I hope this note bears testimony to the honor placed on his dedication, scholarly contribution, and leadership in the field. Dr. Hare Krishna Tiwari was corresponding author of the paper upto the day of its acceptance. Placing of his name, not as a corresponding author in the final version is purely for technical reasons.

## Data Availability Statement

The datasets presented in this study can be found in online repositories. The names of the repository/repositories and accession number(s) can be found in the article/ Supplementary Material.

## Author Contributions

HT designed the study, supervised the study, and helped in manuscript preparation. AS performed the experiments, analyzed the data, and prepared the manuscript. SD and SS analyzed the data and prepared the manuscript. VG helped in the sample collection, survey, and lab experiments. SK and IN helped in manuscript preparation. YL helped in the selection of study area and official documentation regarding the study. All the authors contributed to the article and approved the submitted version.

## Conflict of Interest

The authors declare that the research was conducted in the absence of any commercial or financial relationships that could be construed as a potential conflict of interest.

## Supplementary Material

Supplementary File 1 Springs Detail.

Supplementary File 2 Flow chart of the study design.

Supplementary File 3 Colony and cell morphology of bacterial isolates on different culture media.

Supplementary File 4 Standard biochemical test used for the identification of bacterial isolates.

Supplementary File 5 16S rRNA gene sequencing result and accession number.

Supplementary File 6 Antibiotic resistance pattern of bacterial isolates of spring water of Sikkim (in percentage).

## References

[B1] Abbasi E. Abtahi H. Belkum A. Van Ghaznavi-Rad E. (2019). Multidrug-resistant *Shigella* infection in pediatric patients with diarrhea from central Iran. *Infect. Drug Resist.* 12 1535–1544. 31239729 10.2147/IDR.S203654 PMC6559769

[B2] Adegoke A. A. Madu C. E. Aiyegoro O. A. Stenström T. A. Okoh A. I. (2020). Antibiogram and beta-lactamase genes among cefotaxime resistant E. coli from wastewater treatment plant. *Antimicr. Resist. Infect. Contr.* 9 1–12. 10.1186/s13756-020-0702-4 32164766 PMC7068970

[B3] Adzitey F. (2020). Incidence and antimicrobial susceptibility of *Escherichia coli* isolated from beef (meat muscle, liver and kidney) samples in Wa Abattoir, Ghana. *Cogent Food Agricult.* 6:1718269 10.1080/23311932.2020.1718269

[B4] Adzitey F. Nafisah S. Haruna A. (2015). Antibiotic Susceptibility of *Escherichia coli* Isolated from some Drinking Water Sources in Tamale Metropolis of Ghana. *Curr. Res. Bacteriol.* 8 34–40. 10.3923/crb.2015.34.40

[B5] Al-Bahry S. Al-Sharji N. Yaish M. Al-Musharafi S. Mahmoud I. (2016). Diversity of Tetracycline Resistant Genes in from Human and Environmental Sources. *Open Biotechnol. J.* 10 289–300. 10.2174/1874070701610010289

[B6] Almakki A. Jumas-Bilak E. Marchandin H. Licznar-Fajardo P. (2019). Antibiotic resistance in urban runoff. *Sci. Total Environ.* 667 64–76. 10.1016/j.scitotenv.2019.02.183 30826682

[B7] Amador P. Fernandes R. Prudêncio C. Duarte I. (2019). Prevalence of Antibiotic Resistance Genes in Multidrug-Resistant *Enterobacteriaceae* on Portuguese Livestock Manure. *Antibiotics* 8:23. 10.3390/antibiotics8010023 30871244 PMC6466527

[B8] Amaya E. Reyes D. Paniagua M. Calderón S. Rashid M. U. Colque P. (2012). Antibiotic resistance patterns of *Escherichia coli* isolates from different aquatic environmental sources in León, Nicaragua. *Clin. Microbiol. Infecti.* 18 E347–54. 10.1111/j.1469-0691.2012.03930.x 22738232

[B9] Amer M. M. Mekky H. M. Amer A. M. Fedawy H. S. (2018). Antimicrobial resistance genes in pathogenic *Escherichia coli* isolated from diseased broiler chickens in Egypt and their relationship with the phenotypic resistance characteristics. *Veter. World* 11 1082–1088. 10.14202/vetworld.2018.1082-1088 30250367 PMC6141278

[B10] American Public Health Association (1999). “Detection of pathogenic bacteria, standard methods for the examination of water and wastewater,” in *American Water Works Association and the Water Environment Federation 20th ed*, eds Rice E. W. Baird R. B. Eaton A. D. Clesceri L. S. (Washington, DC: American Public Health Association).

[B11] Anand T. Bera B. C. Vaid R. K. Barua S. Riyesh T. Virmani N. (2016). Abundance of antibiotic resistance genes in environmental bacteriophages. *J. Gener. Virol.* 97 3458–3466. 10.1099/jgv.0.000639 27902329

[B12] Annavajhala M. K. Gomez-Simmonds A. Uhlemann A. C. (2019). Multidrug-resistant *Enterobacter cloacae* complex emerging as a global, diversifying threat. *Front. Microbiol.* 10:1–8. 10.3389/fmicb.2019.00044 30766518 PMC6365427

[B13] Bain R. Cronk R. Hossain R. Bonjour S. Onda K. Wright J. (2014). Global assessment of exposure to faecal contamination through drinking water based on a systematic review. *Trop. Med. Int. Health* 19 917–927. 10.1111/tmi.12334 24811893 PMC4255778

[B14] Bandyopadhyay S. Lodh C. Rahaman H. Bhattacharya D. Bera A. K. Ahmed F. A. (2012). Characterization of shiga toxin producing (STEC) and enteropathogenic *Escherichia coli* (EPEC) in raw yak (Poephagus grunniens) milk and milk products. *Res. Veter. Sci.* 93 604–610. 10.1016/j.rvsc.2011.12.011 22226073

[B15] Baquero F. Martínez J. L. Cantón R. (2008). Antibiotics and antibiotic resistance in water environments. *Curr. Opin. Biotechnol.* 19 260–265. 10.1016/j.copbio.2008.05.006 18534838

[B16] Baral N. Nayak N. Shrestha R. Parajuli R. Hamal D. Bhatta D. R. (2018). Hafnia Alvei Bacteremia Following Bronchopneumonia in an Eleven Month Old Child: a Case Report From a Tertiary Care Hospital in Nepal. *Int. J. Adv. Life Sci. Res.* 1 22–25. 10.31632/ijalsr.2018v01i02.004

[B17] Bengtsson-Palme J. Kristiansson E. Larsson D. G. J. (2018). Environmental factors influencing the development and spread of antibiotic resistance. *FEMS Microbiol. Rev.* 42 68–80. 10.1093/femsre/fux053 29069382 PMC5812547

[B18] Bhatt P. Tandel K. Shete V. Rathi K. R. (2015). Burden of extensively drug-resistant and pandrug-resistant Gram-negative bacteria at a tertiary-care centre. *N Microb. N Infect.* 8 166–170. 10.1016/j.nmni.2015.01.003 27257498 PMC4877402

[B19] Biswas K. Paul D. Sinha N. (2015). Prevalence of Multiple Antibiotic-Resistant Coliform Bacteria in the Water of River Ganga. *Front. Environ. Microbiol.* 1 44–46. 10.11648/j.fem.20150103.12

[B20] Bolaji A. S. Akande I. O. Iromini F. A. Adewoye S. O. Opasola A. O. (2011). Antibiotic resistance pattern of bacteria spp isolated from hospital waste water in Ede South Western, Nigeria. *Eur. J. Exper. Biol.* 1 66–71.

[B21] Bueno I. Williams-Nguyen J. Hwang H. Sargeant J. M. Nault A. J. Singer R. S. (2018). Systematic Review: Impact of point sources on antibiotic-resistant bacteria in the natural environment. *Zoon. Publ. Health* 65 162–184e. 10.1111/zph.12426 29205899

[B22] Chen Z. Yu D. He S. Ye H. Zhang L. Wen Y. (2017). Prevalence of antibiotic-resistant *Escherichia coli* in drinking water sources in Hangzhou City. *Front. Microbiol.* 8 1–11. 10.3389/fmicb.2017.01133 28670309 PMC5472731

[B23] CLSI (2014). *M100-S25 Performance Standards for Antimicrobial Susceptibility Testing; Twenty-Fourth International Supplement*, 24th Edn Wayne, PA: Clinical and Laboratory Standards Institute.

[B24] Costa E. C. Arpini C. M. (2016). Antibiotic Sensitivity Profile of Enteric Bacteria Isolated from Beach Waters and Sewage from the Municipality of Vila Velha-ES, Brazil. *J. Bacteriol. Parasitol.* 7 3–7. 10.4172/2155-9597.1000280

[B25] Davies J. Davies D. (2010). Origins and Evolution of Antibiotic Resistance. *Microbiol. Mole. Biol. Rev.* 74 417–433. 10.1128/mmbr.00016-10 20805405 PMC2937522

[B26] Davis R. Brown P. D. (2016). Multiple antibiotic resistance index, fitness and virulence potential in respiratory *Pseudomonas aeruginosa* from Jamaica. *J. Med. Microbiol.* 65 261–271. 10.1099/jmm.0.000229 26860081

[B27] Dehkordi F. S. Yazdani F. Mozafari J. Valizadeh Y. (2014). Virulence factors, serogroups and antimicrobial resistance properties of *Escherichia coli* strains in fermented dairy products. *BMC Res. Notes* 7 1–8. 10.1186/1756-0500-7-217 24708594 PMC3983858

[B28] Diwan V. Hanna N. Purohit M. Chandran S. Riggi E. Parashar V. (2018). Seasonal variations in water-quality, antibiotic residues, resistant bacteria and antibiotic resistance genes of *Escherichia coli* isolates from water and sediments of the Kshipra River in Central India. *Int. J. Environ. Res. Publ. Health* 15 1–16. 10.3390/ijerph15061281 29914198 PMC6024939

[B29] DuPont H. L. Levine M. M. Hornick R. B. Formal S. B. (1989). Inoculum Size in Shigellosis and Implications for Expected Mode of Transmission. *J. Infect. Dis.* 159 1126–1128. 2656880 10.1093/infdis/159.6.1126

[B30] Farmer J. J. Fanning G. R. Davis B. R. Hara C. M. O. Riddle C. Asbury M. A. (1985). *Escherichia fergusonii* and *Enterobacter* taylorae, Two New Species of *Enterobacteriaceae* Isolated from Clinical Specimens. *J. Clin. Microbiol.* 21 77–81. 3968204 10.1128/jcm.21.1.77-81.1985 PMC271579

[B31] Gaastra W. Svennerhoim A.-M. (1996). Colonization factors of Eschericbia coli (ETEC). *Trends Microbiol.* 4 444–452. 8950814 10.1016/0966-842x(96)10068-8

[B32] Gajul S. V. Mohite S. T. Mangalgi S. S. Wavare S. M. Kakade S. V. (2015). *Klebsiella Pneumoniae* in Septicemic Neonates with Special Reference to Extended Spectrum β -lactamase, AmpC, Metallo β -lactamase Production and Multiple Drug Resistance in Tertiary Care Hospital. *J. Labor. Physic.* 7 32–37. 10.4103/0974-2727.151689 25949057 PMC4411807

[B33] Government of India, Ministry of Health & Family Welfare (2019). *National Health Profile (14th Issue): Prepared by Central Bureau of Health Intelligence.* Directorate General of Health Services Ministry of Health & Family Welfare, Government of India Available online at: https://www.cbhidghs.nic.in/showfile.php?lid=1147

[B34] Grenni P. Ancona V. Barra Caracciolo A. (2018). Ecological effects of antibiotics on natural ecosystems: A review. *Microchem. J.* 136 25–39. 10.1016/j.microc.2017.02.006

[B35] Gunduz S. Uludað Altun H. (2018). Antibiotic resistance patterns of urinary tract pathogens in Turkish children. *Glob. Health Res. Policy* 3 1–5. 10.1186/s41256-018-0063-1 29568806 PMC5856228

[B36] Guo J. Li J. Chen H. Bond P. L. Yuan Z. (2017). Metagenomic analysis reveals wastewater treatment plants as hotspots of antibiotic resistance genes and mobile genetic elements. *Water Res.* 123 468–478. 10.1016/j.watres.2017.07.002 28689130

[B37] Guo X. Li J. Yang F. Yang J. Yin D. (2014). Science of the Total Environment Prevalence of sulfonamide and tetracycline resistance genes in drinking water treatment plants in the Yangtze River Delta, China. *Sci. Total Environ.* 493 626–631. 10.1016/j.scitotenv.2014.06.035 24984233

[B38] Guo X. Rao Y. Guo L. Xu H. Lv T. Yu X. (2019). Detection and Genomic Characterization of a *Morganella morganii* Isolate From China That Produces NDM-5. *Front. Microbiol.* 10:1–9. 10.3389/fmicb.2019.01156 31191484 PMC6546717

[B39] Hagiwara S. Murata M. Aoki M. Kaneko M. Oshima K. (2013). Septic shock caused by Klebsiella oxytoca: An autopsy case and a survival case with driving Extracorporeal Membrane Oxygenation. *Hippokratia* 17 171–173. 24376326 PMC3743625

[B40] Haseena M. Malik M. F. Javed A. Arshad S. Asif N. SharonZulfiqar (2017). Water pollution and human health. *Environ. Risk Assess. Remed.* 1 16–19.

[B41] Hassan Rashid M. A. U. Manzoor M. M. Mukhtar S. (2018). Urbanization and its effects on water resources: An exploratory analysis. *Asian J. Water Environ. Pollut.* 15 67–74. 10.3233/AJW-180007

[B42] Hawkey E. W. W. P. M. Penner J. L. Senior B. W. Barton L. J. (1983). Serious nosocomial infection caused by *Morganella morganii* and *Proteus mirabilis* in a cardiac surgery unit. *J. Clin. Microbiol.* 18 5–9. 6350354 10.1128/jcm.18.1.5-9.1983 PMC270734

[B43] Hawkey P. M. Warren R. E. Livermore D. M. McNulty C. A. M. Enoch D. A. Otter J. A. (2018). Treatment of infections caused by multidrug-resistant gram-negative bacteria: Report of the British society for antimicrobial chemotherapy/healthcare infection society/british infection association joint working party. *J. Antimicrob. Chemother.* 73 iii2–iii78. 10.1093/jac/dky027 29514274

[B44] Hemmatinezhad B. Khamesipour F. Mohammadi M. Dehkordi F. S. Mashak Z. (2015). Microbiological Investigation of O-Serogroups, Virulence Factors and Antimicrobial Resistance Properties of Shiga Toxin-Producing *Escherichia Coli* Isolated from Ostrich, Turkey and Quail Meats. *J. Food Safe.* 35 491–500. 10.1111/jfs.12199

[B45] Hemme C. L. Tu Q. Shi Z. Qin Y. Gao W. Deng Y. (2015). Comparative metagenomics reveals impact of contaminants on groundwater microbiomes. *Front. Microbiol.* 6:1–12. 10.3389/fmicb.2015.01205 26583008 PMC4628106

[B46] Ibrahim H. K. Almayah A. A. Issa A. H. (2017). Molecular Detection of Environmental *Morganella morganii* as Histamine Producing Bacteria Molecular Detection of Environmental *Morganella morganii* as Histamine Producing Bacteria. *Donnish J. Med. Medic. Sci.* 4 8–13.

[B47] Igwaran A. Iweriebor B. C. Okoh A. I. (2018). Molecular characterization and antimicrobial resistance pattern of *Escherichia coli* recovered from wastewater treatment plants in Eastern Cape South Africa. *Int. J. Environ. Res. Publ. Health* 15:1237. 10.3390/ijerph15061237 29895735 PMC6025339

[B48] Jabbar Ibrahim I. A. Kareem Hameed T. A. (2015). Isolation, Characterization and Antimicrobial Resistance Patterns of Lactose-Fermenter *Enterobacteriaceae* Isolates from Clinical and Environmental Samples. *Open J. Med. Microbiol.* 05 169–176. 10.4236/ojmm.2015.54021

[B49] Janda J. M. Abbott S. L. (2006). The genus Hafnia: From soup to nuts. *Clin. Microbiol. Rev.* 19 12–28. 10.1128/CMR.19.1.12-28.2006 16418520 PMC1360275

[B50] Kahsay A. G. Muthupandian S. (2016). A review on Sero diversity and antimicrobial resistance patterns of *Shigella* species in Africa, Asia and South America, 2001–2014. *BMC Res. Notes* 9:422. 10.1186/s13104-016-2236-7 PMC5004314 27576729

[B51] Karkman A. Do T. T. Walsh F. Virta M. P. J. (2018). Antibiotic-Resistance Genes in Waste Water. *Trends Microbiol.* 26 220–228. 10.1016/j.tim.2017.09.005 29033338

[B52] Khanna A. Singh N. Aggarwal A. Khanna M. (2012). The antibiotic resistance pattern in citrobacter species: An emerging nosocomial pathogen in a tertiary care hospital. *J. Clin. Diagn. Res.* 6 642–644.

[B53] Krumperman P. H. (1983). Multiple Antibiotic Resistance Indexing of *Escherichia coli* to Identify High-Risk Sources of Fecal Contamination of Foodst. *Appl. Environ. Microbiol.* 46:6. 10.1128/aem.46.1.165-170.1983 PMC239283 6351743

[B54] Kuhnert P. Boerlin P. Frey J. (2000). Target genes for virulence assessment of *Escherichia coli* isolates from water, food and the environment. *FEMS Microbiol. Rev.* 24 107–117. 10640601 10.1111/j.1574-6976.2000.tb00535.x

[B55] Lancelevee J. Bret L. David K. Di Martino P. (2014). Antibiotic Resistance and Adherence Properties of Hafnia alvei Clinical Isolates: A 19-Month Study in the Hospital of Orléans, France. *J. Chemother.* 19 677–681. 10.1179/joc.2007.19.6.677 18230550

[B56] Lee J. J. Sun H. I. Park K. S. Lee J. E. Ahn J. H. Lee S. H. (2010). Novel Variants of the qnrB Gene, qnrB22 and qnrB23,in Citrobacter werkmanii and Citrobacter freundii. *Antimicr. Agents Chemother.* 54 3068–3069. 10.1128/AAC.01339-09 20421404 PMC2897306

[B57] Li D. Yu T. Zhang Y. Yang M. Li Z. Liu M. (2010). Antibiotic resistance characteristics of environmental bacteria from an oxytetracycline production wastewater treatment plant and the receiving river. *Appl. Environ. Microbiol.* 76 3444–3451. 10.1128/AEM.02964-09 20400569 PMC2876458

[B58] Litrenta J. Oetgen M. (2017). Hafnia alvei: A new pathogen in open fractures. *Trauma Case Rep.* 8 41–45. 10.1016/j.tcr.2017.01.019 29644313 PMC5883196

[B59] Liu H. Zhu J. Hu Q. Rao X. (2016). *Morganella morganii*, a non-negligent opportunistic pathogen. *Int. J. Infect. Dis.* 50 10–17. 10.1016/j.ijid.2016.07.006 27421818

[B60] Liu L. Lan R. Liu L. Wang Y. Zhang Y. Wang Y. (2017). Antimicrobial resistance and cytotoxicity of Citrobacter spp. in Maanshan Anhui Province, China. *Front. Microbiol.* 8 1–12. 10.3389/fmicb.2017.01357 28775715 PMC5518651

[B61] Loya M. K. Walsh J. (2015). A Case of Community Acquired Pneumonia Caused by Hafnia alvei Developing Into an Empyema. *Chest* 148:101A 10.1378/chest.2223426

[B62] Lu J. Tian Z. Yu J. Yang M. Zhang Y. (2018). Distribution and Abundance of Antibiotic Resistance Genes in Sand Settling Reservoirs and Drinking Water Treatment Plants across the Yellow River, China. *Water* 10 1–12. 10.3390/w1003024630079254

[B63] Luna-Guevara J. J. Arenas-Hernandez M. M. P. Martínez de la Peña C. Silva J. L. Luna-Guevara M. L. (2019). The Role of Pathogenic E. coli in Fresh Vegetables: Behavior, Contamination Factors, and Preventive Measures. *Int. J. Microbiol.* 2019:2894328. 10.1155/2019/2894328 31885595 PMC6899298

[B64] Madhavan A. Balakrishnan So Vasudevapanicker J. (2018). Antibiotic susceptibility pattern of *Shigella* isolates in a tertiary healthcare center. *J. Labor. Phys.* 10 140–144. 10.4103/JLP.JLP PMC5896178 29692577

[B65] Mahapatra A. Mahapatra S. Mahapatra A. (2009). *Escherichia fergusonii*: An emerging pathogen in South Orissa. *Indian J. Med. Microbiol.* 23 204–208. 10.4103/0255-0857.16598 16100434

[B66] Malau E. Ford R. Valcanis M. Jennison A. V. Mosse J. Bean D. (2018). Antimicrobial sensitivity trends and virulence genes in *Shigella* spp. from the Oceania region. *Infect. Genet. Evolut.* 64 52–56. 10.1016/j.meegid.2018.06.015 29906636

[B67] Meng L. Liu H. Lan T. Dong L. Hu H. Zhao S. (2020). Antibiotic Resistance Patterns of *Pseudomonas* spp. Isolated From Raw Milk Revealed by Whole Genome Sequencing. *Front. Microbiol.* 11:1005. 10.3389/fmicb.2020.01005 32655503 PMC7326020

[B68] Minnullina L. Pudova D. Shagimardanova E. Shigapova L. Sharipova M. Mardanova A. (2019). Comparative Genome Analysis of Uropathogenic *Morganella morganii* Strains. *Front. Cell. Infect. Microbiol.* 9 1–14. 10.3389/fcimb.2019.00167 31231616 PMC6558430

[B69] Mishra M. Arukha A. P. Patel A. K. Behera N. Mohanta T. K. Dhananjay Yadav. (2018). Multi-Drug Resistant Coliform: Water Sanitary Standards and Health Hazards. *Front. Micro.* 9 1–8. 10.3389/fphar.2018.00311 29946253 PMC6005870

[B70] Mitali D. Mishra S. S. Tripathy R. C. Dwivedi B. K. (2018). Occurrence of Multiple Antibiotic Resistant E. coli on Surface Water of River Ganga at Allahabad, Uttar Pradesh, India. *Oceanogr. Fisher. Open Access J.* 8:555730 10.19080/ofoaj.2018.08.555730

[B71] Momtaz H. Dehkordi F. S. Hosseini M. J. Sarshar M. Heidari M. (2013). Serogroups, virulence genes and antibiotic resistance in Shiga toxin-producing *Escherichia coli* isolated from diarrheic and non-diarrheic pediatric patients in Iran. *Gut Pathogens* 5 1–10. 10.1186/1757-4749-5-39 24330673 PMC3866933

[B72] Momtaz H. Safarpoor Dehkordi F. Taktaz T. Rezvani A. Yarali S. (2012). Shiga toxin-producing *Escherichia coli* isolated from bovine mastitic milk: serogroups, virulence factors, and antibiotic resistance properties. *Scientif. World J.* 2012:618709. 10.1100/2012/618709 23213293 PMC3507047

[B73] Monica H. Dias Y. Pai M. Raviglione M. C. (2018). Antibiotic-resistant *Enterobacteriaceae* in healthy gut flora: A report from north Indian semiurban community. *Indian J. Med. Res.* 147 217–220. 10.4103/ijmr.IJMR 31219094 PMC6563735

[B74] Morgan H. D. R. Oxon M. A. Eng M. R. C. S. Lond L. R. C. P. Camb D. P. H. (1906). Upon The Bacteriology of the Summer Diarrhoea of Infants. *Br. J.* 1 908–912. 10.1136/bmj.1.2364.908 20762625 PMC2381076

[B75] Morris S. Cerceo E. (2020). Trends, epidemiology, and management of multi-drug resistant gram-negative bacterial infections in the hospitalized setting. *Antibiotics* 9 1–20. 10.3390/antibiotics9040196 32326058 PMC7235729

[B76] Muringani B. N. (2017). Antibiotic Susceptibility Patterns of E. Coli Isolated from Water and Stool Samples in Mthata Region Eastern Cape Province of South Africa. *J. Bacteriol. Mycol. Open Acces.* 3 264–265. 10.15406/jbmoa.2016.03.00067

[B77] Murray P. R. Baron E. J. Jorgensen J. H. Landry M. L. Pfaller M. A. (eds) (2017). *Mannual of Clinical Microbiology. 9th Editio.* Washington, D. C: American Society for Microbiology.

[B78] Mustapha A. Imir T. (2019). Detection of Multidrug-Resistance Gram-Negative Bacteria from Hospital Sewage in North East, Nigeria. *Front. Environ. Microbiol.* 5:1 10.11648/j.fem.20190501.11

[B79] Na G. Lu Z. Gao H. Zhang L. Li Q. Li R. (2018). The effect of environmental factors and migration dynamics on the prevalence of antibiotic-resistant *Escherichia coli* in estuary environments. *Scientif. Rep.* 8 1–9. 10.1038/s41598-018-20077-x 29374235 PMC5786026

[B80] Nahar N. Rashid R B. (2018). Phylogenetic Analysis of the Antibiotic Resistance Genes in *Salmonella* Species in silico. *J. Bioanalys. Biomed.* 10 1–12. 10.4172/1948-593x.1000198

[B81] Obayiuwana A. Ibekwe A. M. (2020). Antibiotic resistance genes occurrence in wastewaters from selected pharmaceutical facilities in Nigeria. *Water* 12:1897 10.3390/w12071897

[B82] Obayiuwana A. Ogunjobi A. Yang M. Ibekwe M. (2018). Characterization of bacterial communities and their antibiotic resistance profiles in wastewaters obtained from pharmaceutical facilities in lagos and Ogun states, Nigeria. *Int. J. Environ. Res. Publ. Health* 15:1365. 10.3390/ijerph15071365 29966226 PMC6069043

[B83] Obi C. L. Green E. Bessong P. O. Villiers B. De Hoosen A. A. (2004). Gene encoding virulence markers among *Escherichia coli* isolates from diarrhoeic stool samples and river sources in rural Venda communities of South Africa. *Water SA* 30 37–42.

[B84] Pal M. Ayele Y. Hadush A. Panigrahi S. Jadhav V. J. (2018). Air & Water Borne Diseases Public Health Hazards Due to Unsafe Drinking Water. *Air Water Borne Dis.* 7 1–6. 10.4172/2167-7719.1000138

[B85] Parin U. Kirkan S. Arslan S. S. Yuksel H. T. (2018). Molecular identification and antimicrobial resistance of *Escherichia fergusonii* and *Escherichia coli* from dairy cattle with diarrhoea. *Veter. Med.* 63 110–116. 10.17221/156/2017-VETMED

[B86] Praveenkumarreddy Y. Akiba M. Guruge K. S. Balakrishna K. Vandana K. E. Kumar V. (2020). Occurrence of antimicrobial-resistant *escherichia coli* in sewage treatment plants of south India. *J. Water Sanit. Hygiene Devel.* 10 48–55. 10.2166/washdev.2020.051

[B87] Pruss-Ustun A. Bos R. Gore F. Bartram J. (2008). *Safer water, better health: costs, benefits and sustainability of interventions to protect and promote health.* Spain: World Health Organization.

[B88] Qadri F. Svennerholm A.-M. Faruque A. S. Sack R. B. (2005). Enterotoxigenic *Escherichia coli* in Developing Countries: Epidemiology, Microbiology, Clinical Features, Treatment, and Prevention. *Clin. Microbiol. Rev.* 18 465–483. 10.1128/CMR.18.3.465 16020685 PMC1195967

[B89] Ram S. Vajpayee P. Shanker R. (2007). Prevalence of multi-antimicrobial-agent resistant shiga toxin and enterotoxin producing *Escherichia coli* in surface waters of river Ganga. *Environ. Sci. Technol.* 41 7383–7388. 10.1021/es0712266 18044515

[B90] Ram S. Vajpayee P. Tripathi U. Singh R. L. Seth P. K. Shanker R. (2008). Determination of antimicrobial resistance and virulence gene signatures in surface water isolates of *Escherichia coli*. *J. Appl. Microbiol.* 105 1899–1908. 10.1111/j.1365-2672.2008.03879.x 18713280

[B91] Ranganathan S. Doucet M. Grassel C. L. Delaine-elias B. Zachos N. C. Barry M. (2019). Evaluating *Shigella* flexneri Pathogenesis in the Human Enteroid Model. *Infect. Immun.* 87 740–718e. 10.1128/IAI.00740-18 PMC6434113 30642900

[B92] Ranjbar R. Masoudimanesh M. Dehkordi F. S. Jonaidi-Jafari N. Rahimi E. (2017). Shiga (Vero)-toxin producing *Escherichia coli* isolated from the hospital foods virulence factors, o-serogroups and antimicrobial resistance properties. *Antimicrob. Resist. Infect. Contr.* 6 1–11. 10.1186/s13756-016-0163-y 28074125 PMC5219770

[B93] Ranjbar R. Safarpoor Dehkordi F. Sakhaei Shahreza M. H. Rahimi E. (2018). Prevalence, identification of virulence factors, O-serogroups and antibiotic resistance properties of Shiga-toxin producing *Escherichia coli* strains isolated from raw milk and traditional dairy products. *Antimicrob. Resist. Infect. Contr.* 7 1–11. 10.1186/s13756-018-0345-x 29686859 PMC5902837

[B94] Reza R. Ali S. Farhad Safarpoor D. (2019). Prevalence of Antibiotic Resistance and Distribution of Virulence Factors in the Shiga Toxigenic *Escherichia coli* Recovered from Hospital Food - ProQuest. *Jundishapur J. Microbiol.* 12 1–8.

[B95] Robicsek A. Strahilevitz J. Sahm D. F. Jacoby G. A. Hooper D. C. (2006). qnr prevalence in ceftazidime-resistant *Enterobacteriaceae* isolates from the United States. *Antimicrob. Agents Chemother.* 50 2872–2874. 10.1128/AAC.01647-05 16870791 PMC1538681

[B96] Sambrook J. F. Russell D. W. (2002). *Molecular Cloning: A Laboratory Manual Sambrook*, 3rd Edn New York: Cold Spring Harbor Laboratory Press.

[B97] Sanderson C. E. Fox J. T. Dougherty E. R. Cameron A. D. S. Alexander K. A. (2018). The changing face of water: A dynamic reflection of antibiotic resistance across landscapes. *Front. Microbiol.* 9 1–13. 10.3389/fmicb.2018.01894 30237787 PMC6135886

[B98] Sandhu R. Aggarwal A. Sayal P. Kumar S. (2019). Intestinal carriage of drug-resistant Gram-negative bacteria belonging to family *Enterobacteriaceae* in children aged 3–14 years: An emerging threat. *Int. J. Health Allied Sci.* 8 108–115. 10.4103/ijhas.IJHAS

[B99] Savino F. Cordisco L. Tarasco V. Calabrese R. Palumeri E. Matteuzzi D. (2009). Molecular identification of coliform bacteria from colicky breastfed infants. *Acta Paediatr. Int. J. Paediatr.* 98 1582–1588. 10.1111/j.1651-2227.2009.01419.x 19604166

[B100] Savino F. Cordisco L. Tarasco V. Locatelli E. Di Gioia D. Oggero R. (2011). Antagonistic effect of *Lactobacillus* strains against gas-producing coliforms isolated from colicky infants. *BMC Microbiol.* 11:157. 10.1186/1471-2180-11-157 21718486 PMC3224137

[B101] Sayah R. S. Kaneene J. B. Johnson Y. Miller R. (2005). Patterns of Antimicrobial Resistance Observed in *Escherichia coli* Isolates Obtained from Domestic- and Wild-Animal Fecal Samples, Human Septage, and Surface Water. *Appl. Environ. Microbiol.* 71 1394–1404. 10.1128/AEM.71.3.1394 15746342 PMC1065171

[B102] Shakya P. Barrett P. Diwan V. Marothi Y. Shah H. Chhari N. (2013). Antibiotic resistance among *Escherichia coli* isolates from stool samples of children aged 3 to 14 years from Ujjain, India. *BMC Infect. Dis.* 13 1–6. 10.1186/1471-2334-13-477 24124728 PMC3853101

[B103] Sidhu J. P. S. Ahmed W. Hodgers L. Toze S. (2013). Occurrence of virulence genes associated with diarrheagenic pathotypes in *Escherichia coli* isolates from surface water. *Appl. Environ. Microbiol.* 79 328–335. 10.1128/AEM.02888-12 23124225 PMC3536071

[B104] Simkhada P. Lamichhane S. Subedi S. Shrestha U. T. (2016). Bacteriological Profile and Antibiotic Susceptibility Pattern of Blood Culture Isolates from Patients Visiting Tertiary Care Bacteriological Profile and Antibiotic Susceptibility Pattern of Blood Culture Isolates from Patients Visiting Tertiary Care H. *Glob. J. Inc* 16 1–9.

[B105] Singh A. K. Das S. Singh S. Gajamer V. R. Pradhan N. Lepcha Y. D. (2018b). Prevalence of antibiotic resistance in commensal *Escherichia coli* among the children in rural hill communities of northeast India. *PLoS One* 13:e0199179. 10.1371/journal.pone.0199179 29912980 PMC6005495

[B106] Singh A. K. Das S. Singh S. Gajamer V. Nilu P. (2018a). *First report on Bacterial Diversity of Potable Spring water of Indian Himalayan Region. BioRxiv* [Preprint].

[B107] Singh A. K. Das S. Singh S. Pradhan N. Kumar S. Gajamer V. R. (2019). Physicochemical Parameters and Alarming Coliform Count of the Potable Water of Eastern Himalayan State Sikkim: An Indication of Severe Fecal Contamination and Immediate Health Risk. *Front. Publ. Health* 7 1–17. 10.3389/fpubh.2019.00174 31355173 PMC6636254

[B108] Singh L. Cariappa M. P. Kaur M. (2016). Klebsiella oxytoca: An emerging pathogen? *Med. J. Arm. Forces India* 72 S59–S61. 10.1016/j.mjafi.2016.05.002 28050072 PMC5192185

[B109] Singh N. K. Bezdan D. Checinska Sielaff A. Wheeler K. Mason C. E. Venkateswaran K. (2018c). Multi-drug resistant *Enterobacter* bugandensis species isolated from the International Space Station and comparative genomic analyses with human pathogenic strains. *BMC Microbiol.* 18 1–13. 10.1186/s12866-018-1325-2 30466389 PMC6251167

[B110] Skariyachan S. Mahajanakatti A. B. Grandhi N. J. Prasanna A. Sen B. Sharma N. (2015). Environmental monitoring of bacterial contamination and antibiotic resistance patterns of the fecal coliforms isolated from Cauvery River, a major drinking water source in Karnataka. *India Environ. Monitor. Assess.* 187 187–279. 10.1007/s10661-015-4488-4 25896199

[B111] Stange C. Sidhu J. P. S. Tiehm A. Toze S. (2016). Antibiotic resistance and virulence genes in coliform water isolates. *Int. J. Hygiene Environ. Health* 219 823–831. 10.1016/j.ijheh.2016.07.015 27497615

[B112] Subbiah M. Caudell M. A. Mair C. Davis M. A. Matthews L. Quinlan R. J. (2020). Antimicrobial resistant enteric bacteria are widely distributed amongst people, animals and the environment in Tanzania. *Nat. Commun.* 11:228. 10.1038/s41467-019-13995-5 31932601 PMC6957491

[B113] Tagoe D. N. A. Nyarko H. Arthur S. A. Birikorang E. (2011). A study of antibiotic susceptibility pattern of bacteria isolates in sachet drinking water sold in the cape cost metropolis of Ghana. *Res. J. Microbiol.* 6 153–158.

[B114] Tambe S. Kharel G. Arrawatia M. L. Kulkarni H. Mahamuni K. Ganeriwala A. K. (2012). Reviving dying springs: climate change adaptation experiments from the Sikkim Himalaya. *Mount. Res. Devel.* 32 62–72. 10.1659/MRD-JOURNAL-D-11-00079.1 30326609

[B115] Tambe S. Kharel G. Subba S. Arrawatia M. L. (2013). Rural water security in the Sikkim Himalaya: status, initiatives and future strategy. *Conf. Paper India Mount. Initiat.* 32 62–72.

[B116] Tobias J. Vutukuru S. R. (2012). Simple and rapid multiplex PCR for identification of the main human diarrheagenic *Escherichia coli*. *Microbiol. Res.* 167 564–570. 10.1016/j.micres.2011.11.006 22192837

[B117] Vaz-Moreira I. Egas C. Nunes O. C. Manaia C. M. (2013). Bacterial diversity from the source to the tap: A comparative study based on 16S rRNA gene-DGGE and culture-dependent methods. *FEMS Microbiol. Ecol.* 83 361–374. 10.1111/1574-6941.12002 22938591

[B118] Vital P. G. Zara E. S. Paraoan C. E. M. Dimasupil M. A. Z. Abello J. J. M. Santos I. T. G. (2018). Antibiotic Resistance and Extended-Spectrum Beta-Lactamase Production of *Escherichia coli* Isolated from Irrigation Waters in Selected Urban Farms in Metro Manila, Philippines. *Water* 10 1–11. 10.3390/w1005054830079254

[B119] Vubil D. Balleste-delpierre C. Mabunda R. Acácio S. Garrine M. Nhampossa T. (2018). Antibiotic resistance and molecular characterization of *shigella* isolates recovered from children aged less than 5 years in Manhiça, Southern Mozambique. *Int. J. Antimicrob. Agents* 51 881–887. 10.1016/j.ijantimicag.2018.02.005 29448013

[B120] Wang Y. Ma Q. Hao R. Zhang Q. Yao S. Han J. (2019). Antimicrobial resistance and genetic characterization of *Shigella* spp. in Shanxi Province, China, during 2006 – 2016. *BMC Microbiol.* 19:116. 10.1186/s12866-019-1495-6 31142259 PMC6542020

[B121] Woo P. C. Leung P. K. Leung K. W. (2000). Identification by 16S ribosomal RNA gene sequencing of an *Enterobacteriaceae* species from a bone marrow transplant recipient. *Mol. Pathol.* 53 211–215. 10.1136/mp.53.4.211 11040945 PMC1186972

[B122] World Health Organisation (2018). *Fact Sheet: Drinking-Water.* Geneva: World Health Organization.

