# Supplementary material for: Distribution of Antibiotic-Resistant Enterobacteriaceae Pathogens in Potable Spring Water of Eastern Indian Himalayas: Emphasis on Virulence Gene and Antibiotic Resistance Genes in Escherichia coli
Source: Front Microbiol. 2020 Nov 5;11:581072. doi: 10.3389/fmicb.2020.581072 (PMC7674312; doi:10.3389/fmicb.2020.581072)
Supplement: Supplementary File 2 — Flow chart of the study design. [file Table_2.DOCX]

| **Bacteria** | **Colony morphology** | | | **Cell morphology** |
| --- | --- | --- | --- | --- |
|  | **MacConkey Agar** | **Eosin methylene blue agar** | ***Salmonella-Shigella* agar** | **Gram staining** |
| *Escherichia coli* | Flat, dry, pink colonies with a surrounding darker pink area of precipitated bile salts | Purple with black center and green metallic sheen | Slight growth, pink or red color colonies | Gram negative Rod shaped bacteria |
| *Escherichia fergusonii* | Flat, dry, pink colonies with a surrounding darker pink area of precipitated bile salts | Purple with black center and green metallic sheen | Slight growth, pink or red color colonies | Gram negative Rod shaped bacteria |
| *Klebsiella oxytoca* | Pink, mucoid colonies | Purple colonies | Pink color colonies | Gram negative Rod shaped bacteria |
| *Citrobacter freundii* | Pink color colonies | Brown color colonies | Colonies are grey to black centered | Gram negative Rod shaped bacteria |
| *Citrobacter amalonaticus* | Pink color colonies | Brown color colonies | Colonies are grey to black centered | Gram negative Rod shaped bacteria |
| *Morganella morganii* | colorless colonies | purple colonies | colorless colonies | Gram negative Rod shaped bacteria |
| *Hafnia alvei* | colorless colonies | Purple colonies | No growth | Gram negative Rod shaped bacteria |
| *Hafnia paralvei* | colorless colonies | Purple colonies | No growth | Gram negative Rod shaped bacteria |
| *Enterobacter sp.* | Pink color colonies with bile precipitate in the center | Purple colonies | Cream Pink | Gram negative Rod shaped bacteria |
| *Shigella flexneri* | Colorless or transparent colonies | Colorless or transparent colonies | Colorless colonies with without blackening | Gram negative Rod shaped bacteria |
| **Bacteria** | **Colony morphology** | | | **Cell morphology** |
| *Geobacillus sp.* | Light Green color colonies on thio-sulphate citrate Bile Salt agar | No growth | No Growth | Gram positive rod shaped bacteria |
