# Supplementary figures and images for: Distribution of Antibiotic-Resistant Enterobacteriaceae Pathogens in Potable Spring Water of Eastern Indian Himalayas: Emphasis on Virulence Gene and Antibiotic Resistance Genes in Escherichia coli
Source: Front Microbiol. 2020 Nov 5;11:581072. doi: 10.3389/fmicb.2020.581072 (PMC7674312; doi:10.3389/fmicb.2020.581072)

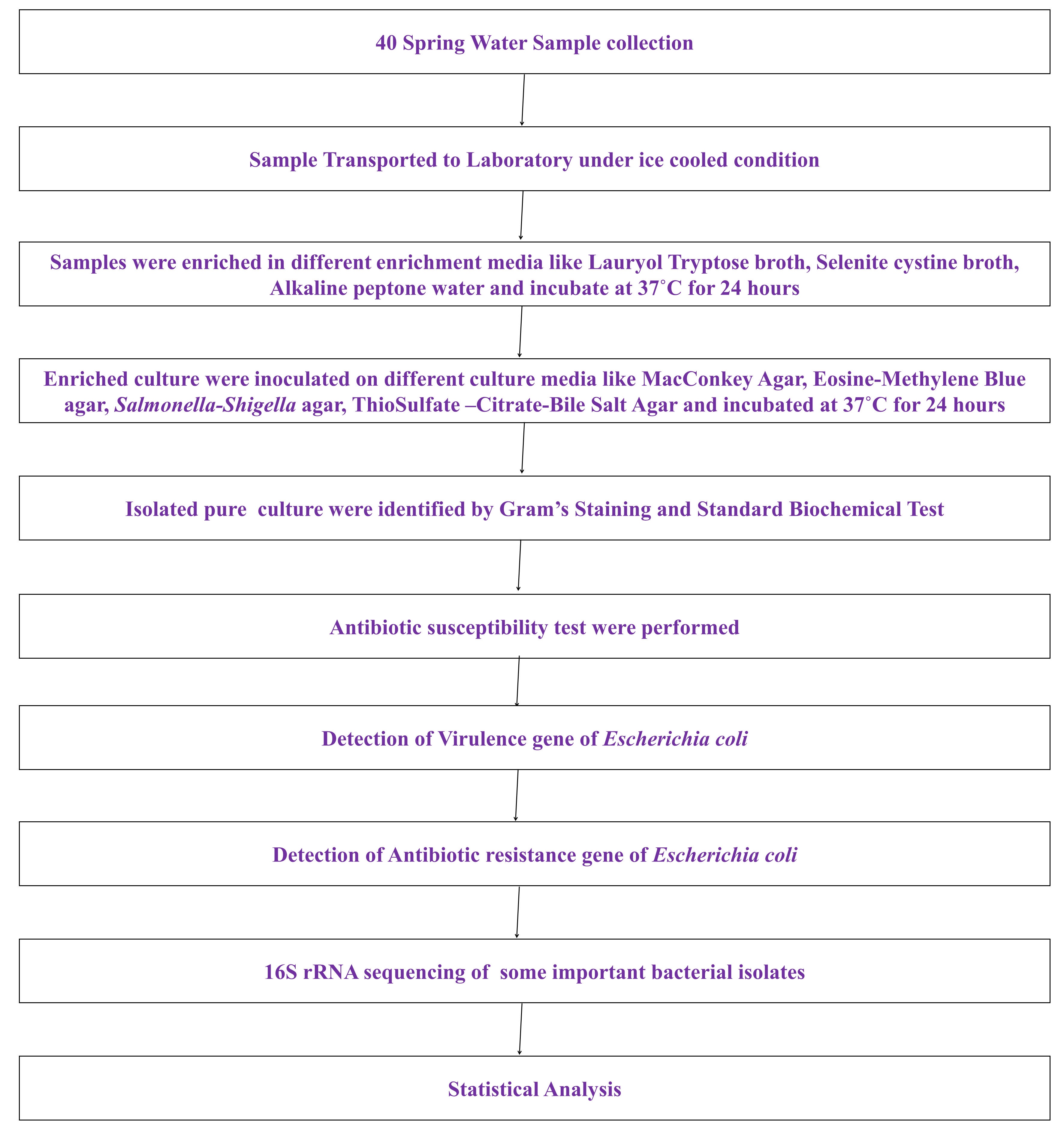

Supplement: Supplementary file 6 [file Image_1.TIF]
